# Pheophorbide A–Mediated Photodynamic Therapy Potentiates Checkpoint Blockade Therapy of Tumor with Low PD–L1 Expression

**DOI:** 10.3390/pharmaceutics14112513

**Published:** 2022-11-18

**Authors:** Qinli Tong, Jiaojiao Xu, Aihua Wu, Chen Zhang, Afeng Yang, Sihang Zhang, Hongzheng Lin, Wei Lu

**Affiliations:** Key Laboratory of Smart Drug Delivery, Ministry of Education & State Key Laboratory of Molecular Engineering of Polymers, Minhang Hospital, School of Pharmacy, Fudan University, 826 Zhangheng Road, Shanghai 201203, China

**Keywords:** programmed death ligand 1 (PD–L1), pheophorbide A, liposomes, photodynamic therapy (PDT), immune checkpoint blockade (ICB), combined therapy

## Abstract

Although the immune checkpoint blockade (ICB) has made a great success in cancer immunotherapy, the overall response rate to the ICB, such as anti–programmed death ligand 1 (PD–L1) therapy, remains only at 20–30%. One major reason is the low expression level of the immune checkpoint in a certain type of tumor cells and its insufficient activation of the host immune system. Herein, we reported a cyclic RGD (cRGD)–modified liposomal delivery system loading the anti–PD–L1 antibody and the photosensitizer pheophorbide A (Pa), allowing a targeting of the low PD–L1 expressing 4T1 mouse breast cancer cells through the recognition of an overexpression of α_v_β_3_ integrin on the tumor cells. The Pa–mediated photodynamic therapy (PDT) elevated the expression of PD–L1 on the tumor cells. PDT, in combination with the anti–PD–L1 therapy, promoted the activation and maturation of dendritic cells as well as the infiltration of cytotoxic T lymphocytes, resulting in the augmented antitumor immune response for the enhanced therapeutic effect. These results demonstrated the combined therapeutic effects of PDT and ICB on the tumor with low PD–L1 levels. Our study suggested that an increase in the PD–L1 expression in tumor cells by PDT would be a promising adjuvant treatment to overcome the ICB irresponsiveness.

## 1. Introduction

With the development of cancer immunotherapy, immune checkpoint inhibitors have attracted a wide attention [1]. Among them, programmed death–ligand 1 (PD–L1) inhibitors have been applied to various types of cancer in clinical treatments [2]. Despite the effectiveness, only less than 30% of patients showed a clinical response to anti–PD–L1 therapy [3]. Existing studies have demonstrated that the efficacy of the PD–L1 blockade therapy relies on the expression level of PD–L1 on the tumors [4]. The insufficient expression of PD–L1 on the tumors leads to the low immune response rate, whereas the increased expression of PD–L1 on the tumors enhances the sensitivity to the PD–L1 inhibitors and improves the prognosis of patients with anti–PD–L1 therapy [5,6]. In addition, in order to evaluate the effectiveness of a particular tumor immune therapy, tumors are usually described as “hot” or “clod” [7]. A “hot” tumor with the high expression of PD–L1 and the enhanced infiltration of the cytotoxic T lymphocytes (CTLs) exhibits a sensitivity to the PD–L1 inhibitors and improves the prognosis of patients with anti–PD–L1 therapy. Comparatively, the “cold” tumor is generally characterized by the lack of a sufficient release of tumor–associated antigens (TAAs) and a sparse infiltration with immune cells, particularly the CTLs, which hinders the clinical application of the PD–L1 inhibitors [8,9,10,11]. Therefore, it is imperative to develop adjuvant therapeutic modalities to improve the efficacy of an anti–PD–L1 treatment in tumors with a low PD–L1 expression.

A number of clinical studies have shown that chemotherapy and radiotherapy synergize the PD–L1 blockade therapy by promoting a T cell infiltration and upregulating the expression of PD–L1 [12,13]. Combining immune checkpoint therapy with photodynamic therapy (PDT) reverses the immunosuppressive cold tumor micro–environment (TME) to a hot TME and stimulates the tumor to respond to the PD–L1 inhibitors [14,15]. PDT stimulates the host immune system by inducing the rapid release of TAAs, which increases the immunogenicity [16]. This process also promotes the maturation of dendritic cells (DCs) and the activation and infiltration of CTLs in the tumor [17,18]. However, only paucity data reported that PDT could increase the expression of PD–L1 in the tissue of the tumors [19]. The effect of PDT on the elevation of the expression of PD–L1 in the tumors with low PD–L1 levels remains to be explored.

Pheophorbide A (Pa) is a second–generation photosensitizer (PS), with a high singlet oxygen quantum yield, and it is a good candidate for PDT of various tumors due to its considerable phototoxicity, but with negligible toxicity to healthy cells in the dark [20,21,22]. However, the poor water solubility, bioavailability and selectivity of Pa limit its wide application [23,24]. Nanoparticulate delivery systems can increase the solubility of PSs and selectively accumulate in the tumor site [25,26]. Liposomes as a dominant type of nanodrug delivery systems used in the drug formulations on the market and have shown great potential to improve the pharmacokinetics and biodistribution of Pa [27]. Furthermore, the tumor accumulation of Pa can be enhanced by modifying the surface of liposomes with the tumor–targeting ligands [28].

Integrins α_v_β_3_, a cell surface receptor related to the initiation, progression and metastasis of solid tumors, is overexpressed on the blood vessels, and in some types of tumors like melanoma, breast and prostate cancer, but not in normal tissues [29]. The peptides containing arginine–glycine–aspartic acid (RGD) have a high selectivity and affinity towards the integrins α_v_β_3_ [30]. Additionally, the cyclic RGD (cRGD) has a higher selectivity and stability than linear RGD [31]. A variety of nanoparticles have been modified with the cRGD to enhance the accumulation and penetration in the tumor through targeting the integrins α_v_β_3_ [32].

**Scheme 1:**
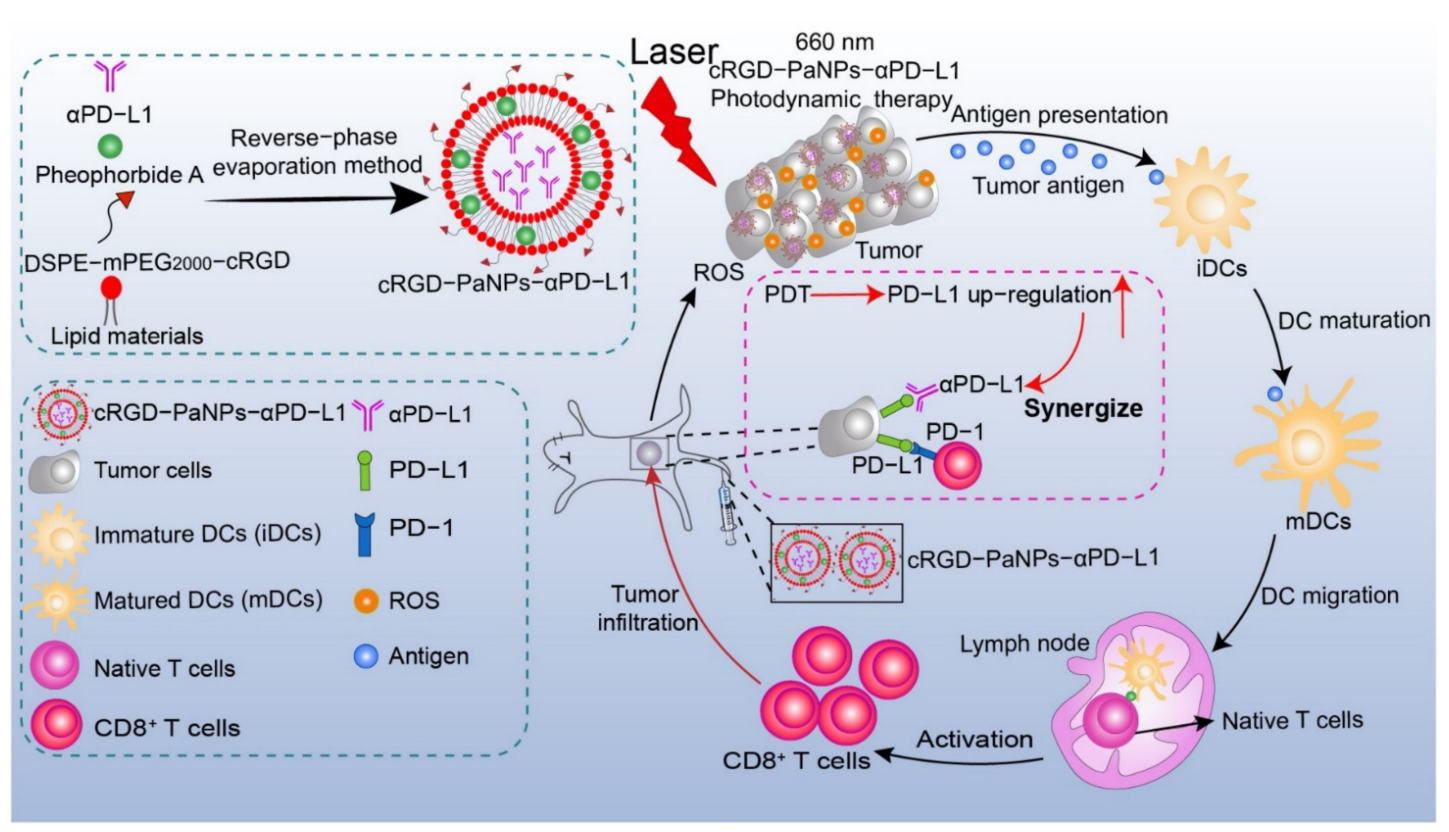
The schematic of the Pa–mediated PDT combined with PD–L1 immune checkpoint blockade therapy for tumor with low PD–L1 expression. The liposomes loading Pa and αPD–L1 (cRGD–PaNPs–αPD–L1) were modified with cRGD in order to target the tumor cells overexpressing α_v_β_3_ integrins. The PD–L1 expression on the tumor cells was up–regulated after PDT, enhancing the sensitivity to αPD–L1. Additionally, PDT effectively promoted the maturation of DCs and increased the activation and infiltration of CTLs in the tumor site to activate the host antitumor immunity.

In this study, we hypothesized that PDT could potentiate the anti–PD–L1 therapy of a tumor with low PD–L1 levels through elevating the PD–L1 expression as well as reversing the immune suppressive microenvironment. We designed an α_v_β_3_ integrin–targeted liposome to load Pa and anti–PD–L1 antibody (αPD–L1) to enhance the accumulation in a low PD–L1 expressing mouse 4T1 breast tumor for combined PDT with PD–L1 blockade therapy (Scheme 1). We found that the cyclic RGD (cRGD)–modified liposomes (cRGD–NPs) improved their tumor targeting effect as well as the solubility and biocompatibility of Pa. The expression of PD–L1 in the tumor cells was up–regulated after the Pa–mediated PDT. The PDT combined with αPD–L1 effectively promoted the maturation of DCs and increased the activation and infiltration of CTLs in the tumor site. The enhanced antitumor therapeutic effect demonstrated that the Pa–mediated PDT is a promising adjuvant treatment to anti–PD–L1 therapy for the low PD–L1 expressing tumor.

## 2. Materials and Methods

### 2.1. Materials

N–(Carbonyl–methoxy polyethylene glycol 2000)–1,2–distearoyl–sn–glycerol–3–phosphoethanolamine, sodium salt (DSPE–mPEG_2000_) and 1,2–dipalmitoyl–sn–glycero–3–phosphocholine (DPPC) were purchased from Advanced Vehicle Technology Pharmaceutical Co., Ltd. (Shanghai, China). DSPE–mPEG_2000_–cRGD was purchased from Hunan Huateng Pharmaceutical Co., Ltd. (Changsha, China). Cholesterol, singlet oxygen sensor green reagent (SOSG), 3–(4,5–dimethyl–2–thiazolyl)–2,5–diphenyl–2H–tetrazolium bromide (MTT), a bicinchoninic acid assay (BCA) protein concentration determination kit, heparin, immunoglobulins (IgG) and fluorescein isothiocyanate (FITC) were purchased from Dalian Meilun Biotechnology Co., Ltd. (Dalian, China). Pa was obtained from AURUM Pharmatech Co., Ltd. (Plano, TX, USA). 4′,6–Diamidino–2–phenylindole (DAPI) and 2′,7′–dichlorofluorescin diacetate (H2–DCFDA) were ordered from Sigma–Aldrich Co., Ltd. (Saint Louis, MO, USA). αPD–L1 (BE0101) was purchased from BioXcell (West Lebanon, NH, USA). Chloroform, methanol and other analytical reagents were purchased form Sinopharm Chemical Reagent Co., Ltd. (Shanghai, China). The cocktail protease inhibitor, TrypLE™ Express Enzyme, flow cytometry antibodies and 1,1′–dioctadecyl–3,3,3′,3′–tetramethylindodicarbocyanine,4–chlorobenzenesulfonate salt (DiD) cell–labeling solution were purchased from Thermo Fisher Scientific (Waltham, MA, USA).

### 2.2. Cells and Animals

The mouse breast tumor cell line 4T1, mouse embryonic fibroblast cells line NIH3T3, mouse melanoma cell line B16F10, mouse Lewis lung cancer (LLC) cell line and mouse colon cancer cell line CT26 were obtained from American Type Culture Collection (Manassas, VA, USA). The cells were cultured in Dulbecco’s modified Eagle’s medium (DMEM, HyClone) or RPMI (1640), supplemented with 10% (*v*/*v*) fetal bovine serum (FBS, HyClone), penicillin (100 µg/mL) and streptomycin (100 µg/mL) at 37 °C and a 5% CO_2_ atmosphere.

BALB/c mice (female, 6–8 weeks) were ordered from Shanghai Lingchang Biological Co., Ltd. (Shanghai, China) and housed under the specific pathogen–free (SPF) conditions with free access to food and water. All animals were allowed to adapt to the environment for at least one week before the experiments.

### 2.3. Preparation and Characterization of the Drug–Loaded Liposomes

The liposomes loading Pa and IgG with (cRGD–PaNPs–IgG) or without (PaNPs–IgG) cRGD modification were prepared by a reverse–phase evaporation method. Briefly, the lipid materials (DSPE–mPEG_2000_, DPPC and cholesterol) were dissolved in 2 mL of chloroform. Pa was dissolved in 1 mL of the mixed organic solvent (chloroform: methanol = 6.5:3.5, *v*/*v*). IgG with or without DSPE–mPEG_2000_–cRGD was dissolved in 1 mL of phosphate–buffered solution (PBS). The volume ratio of the organic phase to aqueous phase was 3:1. The water phase was added into the organic phase. The mixture was sonicated in a water bath for several seconds to form a stable water–in–oil emulsion and then placed on a rotary evaporator to remove the organic solvent. Then, the emulsion was hydrated by adding PBS, followed by an extrusion through a 0.2 and 0.1 µm–sized poly–carbonate membrane sequentially (Whatman, Maidstone, UK).

The particle size, polymer dispersity index (PDI) and zeta–potential of the liposomes were measured by dynamic light scattering (DLS) at 25 °C (Zetasizer Nano ZS90, Malvern Instruments, Malvern, UK). The morphology of the liposomes was observed by a cryo–transmission electron microscope (Cryo–TEM, FEI Tecnai G2 F20, 200 kV, FEI, Hillsboro, OR, USA).

The UV–Vis spectrophotometer was used to quantify the concentration of Pa in the liposomes (670 nm). The drug load efficiency (DL) and encapsulation efficiency (EE) of Pa in the liposomes were calculated using the following equation.

EE% = Weight of the encapsulated Pa/Weight of Pa added × 100%

DL% = Weight of the encapsulated Pa/Weight of liposomes × 100%

The free IgG was removed by ultrafiltration tubes (300 kDa). The concentration of the free IgG was measured by BCA.

EE% = (Total weight of IgG − Free IgG)/Weight of IgG added × 100%

DL% = (Total weight of IgG − Free IgG)/Weight of liposomes × 100%

### 2.4. Stability of PaNPs–IgG or cRGD–PaNPs–IgG

For the stability assay, 1 mL of PaNPs–IgG or cRGD–PaNPs–IgG solution were added into 9 mL of PBS (pH = 7.4) or PBS containing 10% FBS (*v*/*v*), and were stored at 37 °C. The size distribution of the liposomes was measured using DLS at 0, 12, 24, 36 and 48 h, respectively.

### 2.5. In Vitro Drug Release

To evaluate the release profile of Pa, the membrane dialysis bags (3.5 kDa) were loaded with 1 mL of PaNPs–IgG or cRGD–PaNPs–IgG solution (100 µg/mL of Pa), placed in 49 mL of PBS (pH = 7.4) containing 0.5% (*w*/*v*) Tween 80 and incubated at 37 °C (100 rpm). Then, 1 mL of the solution was collected at 0.5, 1, 2, 4, 6, 8, 12 or 24 h. The concentration of Pa was calculated by measuring the fluorescence at λ_ex_ = 415 nm and λ_em_ = 670 nm (FECAN Infinite200 PRO, Bio–Tek Instruments, Rochester, VT, USA).

The drug release from the liposomes loading FITC–labeled IgG was performed in a PBS (pH = 7.4) containing 10% FBS (*v*/*v*). Then, 800 µL of the liposomes (100 µg/mL of IgG) were sealed in dialysis bags (500 kDa) and placed in 29.2 mL of PBS (pH = 7.4) containing 10% FBS (*v*/*v*) at 37 °C (100 rpm). Next, 3 mL of the medium was withdrawn and replaced with the same volume of the fresh medium at 0.25, 0.5, 1, 2, 4, 6, 8, 12 or 24 h. The concentration of the released sample was calculated by measuring the fluorescence of FITC at λ_ex_ = 485 nm and λ_em_ = 520 nm.

### 2.6. The PD–L1 Expression

#### 2.6.1. The PD–L1 Expression in Different Cell Lines

B16F10, LLC, CT26 and 4T1 cells were cultured in 12–well cell plates at a density of 20 × 10^4^ per well, respectively. The cells were harvested by TrypLE™ Express Enzyme and stained with an anti–PD–L1 antibody or its isotype for 30 min at 4 °C. The PD–L1 expression was tested with the flow cytometry (CytoFLEX, Beckman Coulter, Brea, CA, USA).

#### 2.6.2. The PD–L1 Expression in 4T1 Tumor Cells following PDT In Vivo

The DiD–labeled 4T1 cells (200 × 10^4^) were subcutaneously (s.c.) implanted in the right flank of the mice. The tumor–bearing mice were randomly divided into two groups. After the tumor inoculation for 12 d, PDT (660 nm, 100 mW/cm^2^, 10 min) was conducted at 1 h after the intravenous (i.v.) injection with cRGD–PaNPs–IgG (5 mg/kg of Pa, 5 mg/kg of IgG) or PBS. The tumor tissues were harvested after PDT for 12 h and dispersed into single cells by Accumax (Millipore, Billerica, MA, USA) for the PD–L1 staining and flow cytometric analysis.

### 2.7. In Vitro Cellular Uptake

The 4T1 tumor cells were seeded in 24–well cell culture plates at a density of 10 × 10^4^ cells per well for 24 h at 37 °C in 5% CO_2_. The cells were then incubated with PaNPs–IgG or cRGD–PaNPs–IgG containing 0.5, 2, 4 or 8 µM of Pa for 0.5, 1 or 2 h. The cells were harvested and suspended with PBS for the flow cytometric analysis.

To observe the cellular localization of the Pa, the tumor cells were seeded in 12–well cell culture plates at a density of 20 × 10^4^ per well for 24 h. Then, the culture was replaced by DMEM containing PaNPs–IgG or cRGD–PaNPs–IgG (4 µM of Pa) and incubated at 37 °C with 5% CO_2_ for 2 h. After the incubation, the medium was removed and washed twice by cold PBS, fixed with 4% of paraformaldehyde (PFA) for 15 min and stained with DAPI (0.5 µg/mL) for 10 min. The images were recorded with the inverted fluorescence microscope (DMI 4000 B, Leica, Wetzlar, Germany).

### 2.8. Measurement of Reactive Oxygen Species (ROS)

The ROS level produced by the PaNPs–IgG or cRGD–PaNPs–IgG solution was measured with an SOSG kit. Briefly, the liposomal solution containing 5 µM of Pa and 5 µM of SOSG was irradiated with a 660 nm laser at a density of 20 mW/cm^2^ for 5 min (MRL–III–660 D, Cnilaser, Changchun, China). The fluorescence intensity of the SOSG was detected at Ex = 485 nm and Em = 520 nm.

The intracellular ROS level was measured using H2–DCFDA. The tumor cells were seeded at a density of 10 × 10^4^ in 24–well plates for 24 h. PaNPs–IgG and cRGD–PaNPs–IgG (0.4 µM of Pa) were added into the wells for 2 h, respectively. After the incubation, the cells were washed by cold PBS twice. Then, the cells were incubated with H2–DCFDA (10 µM) at 37 °C for 20 min. Free DMEM was added after the cell supernatant was removed. Next, the cells were irradiated by a 660 nm laser for 5 min (20 mW/cm^2^). The cells were harvested by 0.25% trypsin–ethylene diamine tetraacetic acid and analyzed by the flow cytometry. In a parallel experiment, the cells were observed by the fluorescence inverted microscope.

### 2.9. In Vitro Toxicity Analysis

The NIH3T3 cells were seeded in 96–well cell culture plates at a density of 1 × 10^4^ cells per well and incubated at 37 °C in 5% CO_2_ for 24 h. Then, a complete medium was replaced by a DMEM containing PaNPs–IgG or cRGD–PaNPs–IgG with 0.5, 1, 2, 4, 8, 16 or 32 µM of Pa for 24 h. The cell viability was measured by an MTT assay [33].

To analyse the cell–killing effect of PDT in vitro, the 4T1 tumor cells were seeded in 96–well cell culture plates at a density of 1 × 10^4^ cells per well and incubated at 37 °C in 5% CO_2_ for 24 h. Then, the 4T1 cells were incubated with PaNPs–IgG or cRGD–PaNPs–IgG (0.0125, 0.025, 0.05, 0.1, 0.2, 0.4, 0.8, 1.6, 3.2 or 6.4 µM of Pa) for 2 h. After the incubation, the medium was replaced with a fresh DMEM, and the cells were irradiated with a 660 nm laser for 5 min (20 mW/cm^2^). The cells were further cultured for 24 h, followed by an MTT assay. The cell viability was calculated using the following equation.

Cell viability = (A _test_ − A _blank_)/(A _control_ − A _blank_) × 100%

### 2.10. Pharmacokinetics Study

PaNPs–IgG or cRGD–PaNPs–IgG were i.v. injected into normal female BALB/c mice (6–8 weeks, *n* = 3) through the tail veins at the dosage of 5 mg/kg of Pa and 5 mg/kg of IgG. The blood was collected from the tail veins into the heparin tubes at different time points (1, 2, 4, 6, 8, 12 or 24 h). The fluorescence of each sample (20 µL) was analyzed by an in vivo imaging system (IVIS) (Ex = 675 nm, Em = 720 nm). The blood concentration of the Pa was calculated using the following equation:

%ID/mL = (T_blood_ − T_blank_)/0.02 (T_control_ − T_blank_) (1)

where T_blood_, T_blank_ and T_control_ represent the total radiant efficiency of the collected blood, blank blood and control blood (1% of the injection dose), respectively. The pharmacokinetic parameters were calculated using the non–compartmental analysis [34].

### 2.11. Biodistribution Studies

The 4T1 tumor cells (100 × 10^4^) were s.c. injected into the right axilla of the BALB/c mice. The mice were i.v. injected with PaNPs–IgG or cRGD–PaNPs–IgG (5mg/kg of Pa, 5 mg/kg of IgG, *n* = 3) when the tumor volume reached 300 mm^3^. The total radiant efficiency of the tumor sites was detected by an IVIS image system at 1, 2, 4, 6, 8, 12 or 24 h after the injection (Ex = 675 nm, Em = 620 nm).

In a separate experiment, the mice were euthanized at 1 or 12 h after the injection of PaNPs–IgG or cRGD–PaNPs–IgG (5 mg/kg of Pa, 5 mg/kg of IgG, *n* = 3). The heart, liver, spleen, lung, kidney and tumor were harvested for the IVIS imaging. The florescence signals of the major organs and tumors were determined by an average radiant efficiency (Ex = 675 nm, Em = 720 nm).

### 2.12. Evaluation of the Antitumor Effect

The 4T1–bearing mice were randomly divided into four groups and received PBS, αPD–L1, PDT or PDT + αPD–L1 (*n* = 5) at day 9 after the tumor inoculation. For the αPD–L1 treatment group, the mice were intraperitoneally (i.p.) injected with αPD–L1 (100 µg) at days 9, 12 and 15. For the groups of PDT alone or PDT + αPD–L1, the mice were i.v. injected with cRGD–PaNPs–IgG (5 mg/kg of Pa and 100 µg of IgG) or cRGD–PaNPs–αPD–L1 (5 mg/kg of Pa and 100 µg of αPD–L1) at day 9, followed by the laser treatment (660 nm, 100 mW/cm^2^, 10 min) at 1 h post–injection. Afterwards, the mice were i.p. injected with αPD–L1 (100 µg) at days 12 and 15, respectively. The tumor volume and body weight were recorded every three days. The death point was set when the tumor volume reached 2000 mm^3^. The tumor volume was calculated by the following formula:

Tumor volume = length × width^2^/2 (2)

In addition, the tumor sections were stained with a terminal deoxynucleotidyl transferase–mediated dUTP nick–end labeling (TUNEL) assay.

### 2.13. Evaluation of the Anti–Tumor Immunity

The tumors, spleens and draining lymph nodes (DLNs) were collected from the above four groups 5 days after the last injection. The single cell samples were prepared from the tissues, followed by the staining with different fluorescence antibodies. The procedure for both the cellular membrane staining and intracellular staining followed our previously reported method [35]. The data were acquired and analyzed with a CytExpert flow cytometer.

### 2.14. Toxicity Assessment

cRGD–PaNPs–αPD–L1 (5 mg/kg of Pa, 5 mg/kg of αPD–L1) were i.v. injected into healthy BALB/c mice at days 0, 3 and 6, respectively (*n* = 3). The body weight was recorded every three days. The major organs (heart, liver, spleen, lung, kidney, lymph node and thymus) were collected and weighted at day 28. The organ coefficients were calculated using the following equation:

Organ coefficients = organ weight/body weight × 100%

The organs were fixed with 4% of paraformaldehyde to prepare paraffin–embedded slides for hematoxylin and eosin (H&E) staining. The whole blood and serum were collected for the hematology and blood biochemical analysis.

### 2.15. Statistical Analysis

Statistical analysis was performed using Graphpad Prism 8.0. All results were presented as mean ± SD. The results were analyzed by a two–tailed student’s *t*–test between the two groups. A one–way analysis of variance (ANOVA) with Tukey’s comparisons post hoc test or two–way ANOVA with Sidak’s comparisons post hoc test was used for multiple groups. The Kaplan–Meier method was used to analyze the differences in the animals’ survival. * *p* < 0.05, ** *p* < 0.01, *** *p* < 0.001, ^&^ *p* < 0.05, ^&&^ *p* < 0.01 and ^&&&^ *p* < 0.001.

## 3. Results

### 3.1. Characterization of PaNPs–IgG or cRGD–PaNPs–IgG

**Figure 1:**
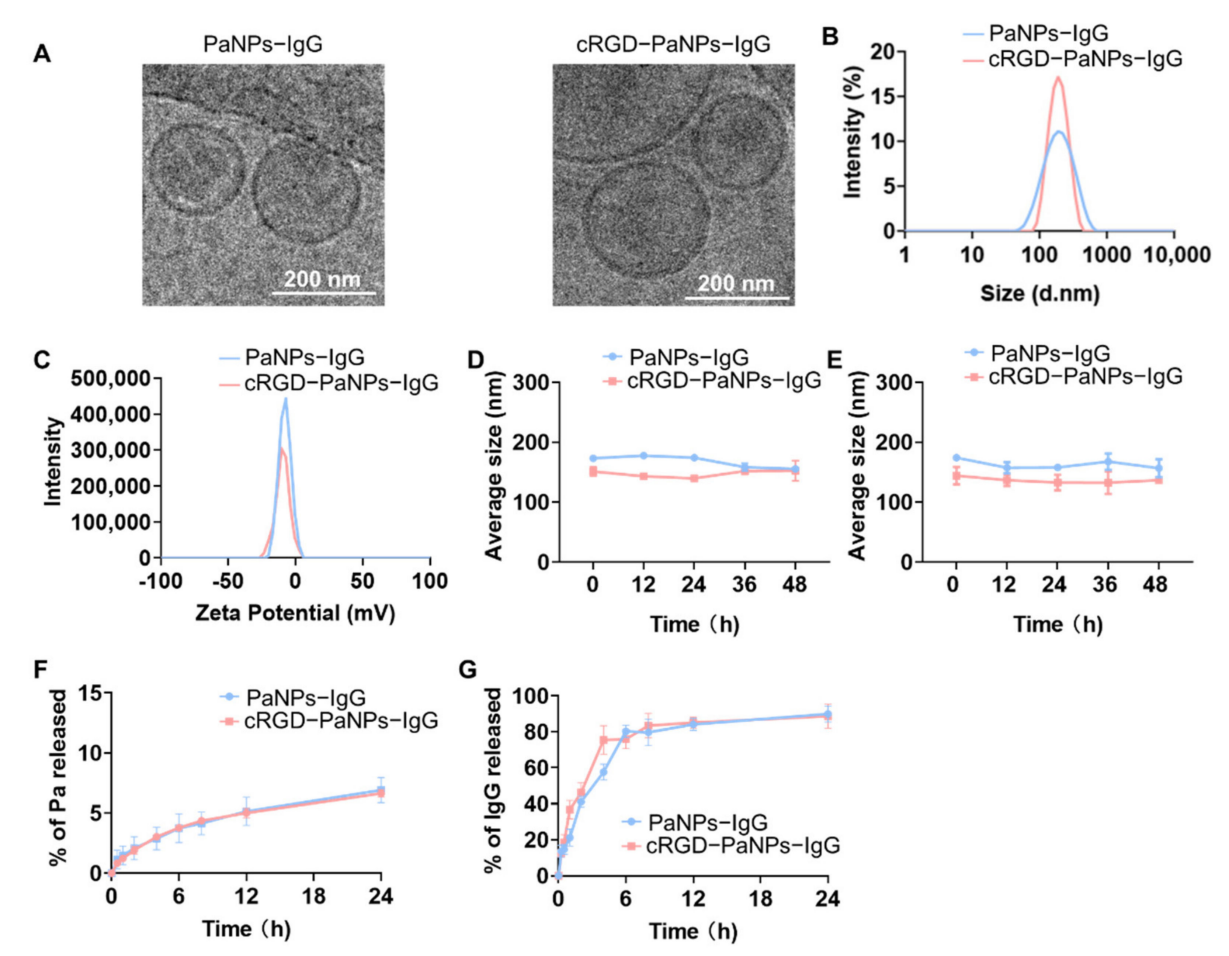
Characterization of PaNPs–IgG or cRGD–PaNPs–IgG. (**A**) The cryo–TEMs, (**B**) size distribution and (**C**) zeta potentials of PaNPs–IgG and cRGD–PaNPs–IgG, respectively. (**D**) Stability of the liposomes in PBS (pH = 7.4) for 48 h. (**E**) Stability of PaNPs–IgG or cRGD–PaNPs–IgG in PBS (pH = 7.4) containing 10% (*v*/*v*) FBS for 48 h. (**F**) Cumulative release of Pa from PaNPs–IgG or cRGD–PaNPs–IgG in PBS (pH = 7.4) containing 0.5% Tween 80 (w/v). (**G**) Cumulative IgG released from PaNPs–IgG or cRGD–PaNPs–IgG in PBS (pH = 7.4) containing 10% FBS (*v*/*v*). Data are presented with mean ± SD (*n* = 3).

TEM depicted PaNPs–IgG and cRGD–PaNPs–IgG in a vesicle shape of 173.70 ± 3.32 nm and 178.00 ± 3.89 nm in diameter, respectively (Figure 1A,B and Supplementary Table S1). The PDI of PaNPs–IgG and cRGD–PaNPs–IgG was 0.19 ± 0.028 and 0.214 ± 0.06, respectively (Supplementary Table S1). The zeta potentials of PaNPs–IgG and cRGD–PaNPs–IgG were −7.88 ± 0.046 mV and −10.20 ± 0.45 mV, respectively (Figure 1C). The EE of Pa and IgG was both above 85% with the DL over 1.6% (Supplementary Tables S2 and S3). These results demonstrated that Pa and IgG were successfully loaded into the liposomal formulations.

### 3.2. Stability of PaNPs–IgG or cRGD–PaNPs–IgG

The stability of the liposomes was investigated in PBS and PBS containing 10% FBS (*v*/*v*), respectively. As shown in Figure 1D,E, the average size of the PaNPs–IgG or cRGD–PaNPs–IgG remained unchanged for 2 days, indicating that both PaNPs–IgG and cRGD–PaNPs–IgG were stable in the physiological environment.

### 3.3. In Vitro Drug Release

The cumulative release rate of Pa from PaNPs–IgG or cRGD–PaNPs–IgG was about 7% at 24 h (Figure 1F). As shown in Figure 1G, the cumulative release rate of the IgG protein from PaNPs–IgG or cRGD–PaNPs–IgG was about 80% at 6 h.

### 3.4. The PD–L1 Expression

**Figure 2:**
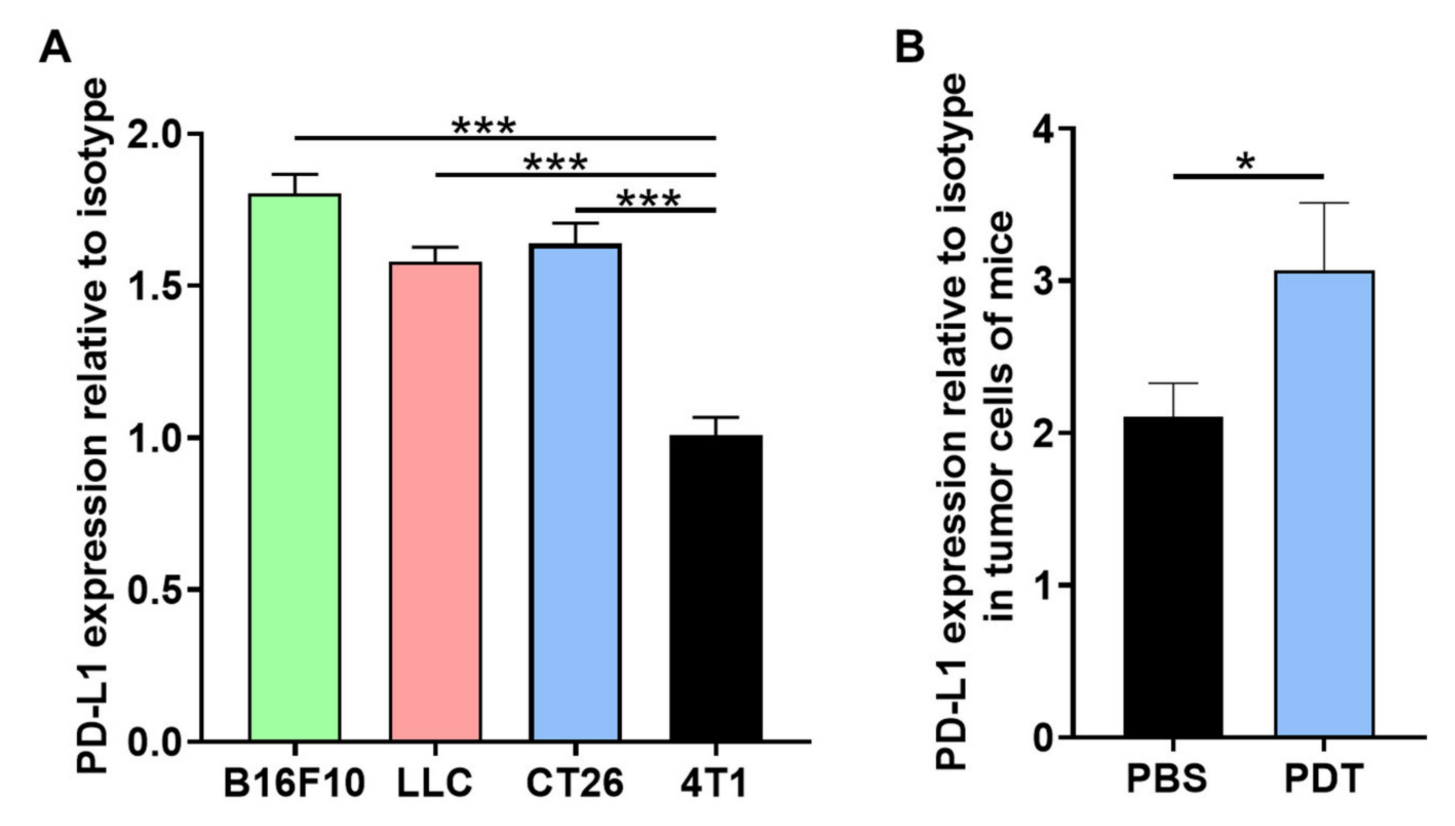
The PD–L1 expression in tumor cells. (**A**) The PD–L1 expression in different tumor cell lines. (**B**) The PD–L1 expression in 4T1 tumor cells in vivo after PDT for 12 h. For the PDT treatment, the mice were i.v. injected with cRGD–PaNPs–IgG (5mg/kg of Pa, 5 mg/kg of IgG). After 12 h, the tumor area received a laser irradiation (660 nm, 100 mW/cm^2^, 10 min). The mice receiving PBS without laser treatment were used as control. Data are presented with mean ± SD (*n* = 3). * *p* < 0.05 and *** *p* < 0.001.

The level of the expression of PD–L1 in different tumor cells was tested with the flow cytometry. As shown in Figure 2A, the relative PD–L1 expression of 4T1 cells to the isotype control was 1.01, indicating that 4T1 tumor cells nearly do not express or express very low levels of PD–L1. On the other hand, the relative expression level in other types of cancer cell lines such as B16F10, LLC and CT26 was 1.80, 1.58 and 1.64–fold, respectively.

In the 4T1 tumor model, the relative expression of PD–L1 in the tumor cells following the PDT treatment was about 1.45–fold of the PBS group (Figure 2B). This result demonstrated that PDT enhanced the expression of PD–L1 in the tumor cells in vivo and would enhance the sensitivity of the tumor cells to αPD–L1. The histograms of the PD–L1 expression were supplied in Supplementary Figure S1.

### 3.5. In Vitro Cellular Uptake

The treatment effect of PDT was dependent on the extent of internalization of photosensitizers. After the treatment with PaNPs–IgG or cRGD–PaNPs–IgG, the fluorescence intensity of Pa in cRGD–PaNPs–IgG was significantly higher than the PaNPs–IgG at various concentrations for a different incubation time (Supplementary Figure S2A), supporting that cRGD–PaNPs–IgG had a stronger tumor target ability than PaNPs–IgG. Additionally, the liposomes were internalized by the 4T1 cells in a concentration– and time–dependent manner. This result was in line with the fluorescence micro–images, showing that the fluorescence signal intensity of cRGD–PaNPs–IgG was stronger in the cRGD–PaNPs–IgG–treated cells with an enhanced cellular uptake than that in the PaNPs–IgG–treated group (Supplementary Figure S2B).

### 3.6. The ROS Generation Ability of the Liposomes

**Figure 3:**
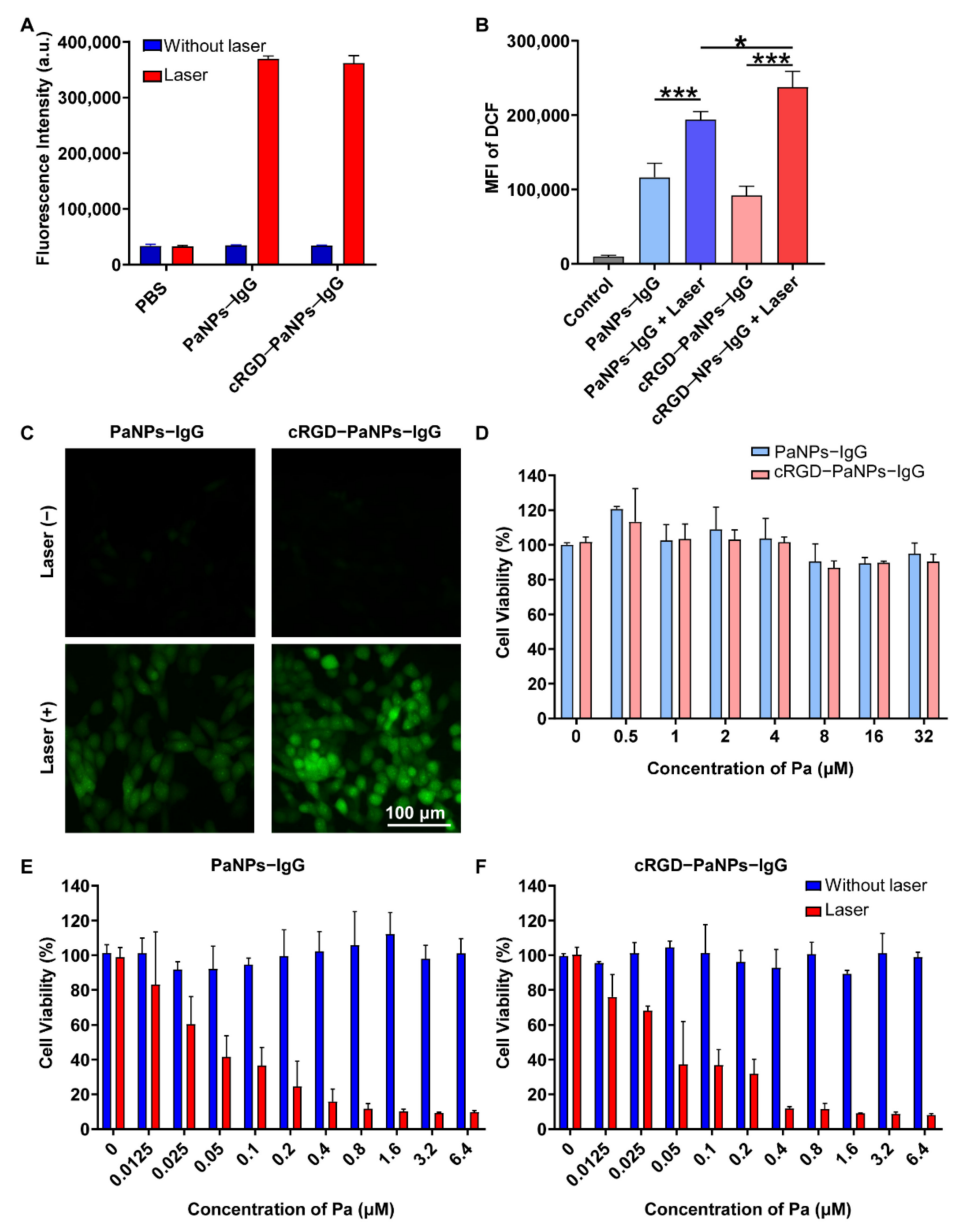
The PDT effect of cRGD–PaNPs–IgG in vitro. (**A**) Singlet oxygen generation of PaNPs–IgG or cRGD–PaNPs–IgG (5 µM of Pa) with or without laser irradiation (660 nm, 20 mW/cm^2^, 5 min). (**B**) Mean fluorescence intensity (MFI) of 4T1 cells with different treatment followed by H2–DCFDA staining, analyzed by flow cytometry. 4T1 cells incubated with H2–DCFDA (10 µM) at 37 °C for 20 min after incubation with the PaNPs–IgG or cRGD–PaNPs–IgG (0.4 µM of Pa) for 2 h. The laser treatment (660 nm, 20 mW/cm^2^, 5 min) was applied at 20 min after incubation with H2–DCFDA (10 µM). (**C**) Fluorescence micrographs of the 4T1 cells following the treatment in (**B**). (**D**) Relative cell ability of NIH3T3 cells after the incubation with PaNPs–IgG or cRGD–PaNPs–IgG for 24 h. (**E**,**F**) Cell ability of 4T1 cells incubated with PaNPs–IgG (**E**) or cRGD–PaNPs–IgG (**F**) with or without laser treatment (660 nm, 20 mW/cm^2^, 5 min) at 2 h post–incubation. The cells were further incubated for 24 h for the MTT assay. Data are mean ± SD (*n* = 3). * *p* < 0.05 and *** *p* < 0.001.

As shown in Figure 3A, a significant amount of ROS was generated in the solution containing either PaNPs–IgG or cRGD–PaNPs–IgG after the laser irradiation. The ROS generation ability of PaNPs–IgG or cRGD–PaNPs–IgG were further measured in 4T1 tumor cells using H2–DCFDA (Figure 3B,C). ROS was significantly increased in either PaNPs–IgG or cRGD–PaNPs–IgG–treated cells following the laser irradiation in comparison with those without a laser treatment. Moreover, a significant increase in the ROS generation in the cRGD–PaNPs–IgG + laser group compared with the PaNPs–IgG + laser group as an indicative of a receptor–mediated endocytosis by cRGD–PaNPs–IgG.

### 3.7. In Vitro Toxicity Analysis

Either PaNPs–IgG or cRGD–PaNPs–IgG did not exhibit a toxicity in the NIH3T3 cells at the incubation concentration of Pa up to 32 µM (Figure 3D), indicating that the liposomes had a good biocompatibility without an observed dark toxicity. In 4T1 tumor cells, the IC_50_ of PaNPs–IgG and cRGD–PaNPs–IgG following the laser treatment were measured as 0.04653 µM and 0.04592 µM, respectively (Figure 3E,F). These data demonstrated that PaNPs–IgG or cRGD–PaNPs–IgG can kill the 4T1 tumor cells at a low concentration with an efficient PDT effect.

### 3.8. Pharmacokinetics and Biodistribution of the Liposomes

**Figure 4:**
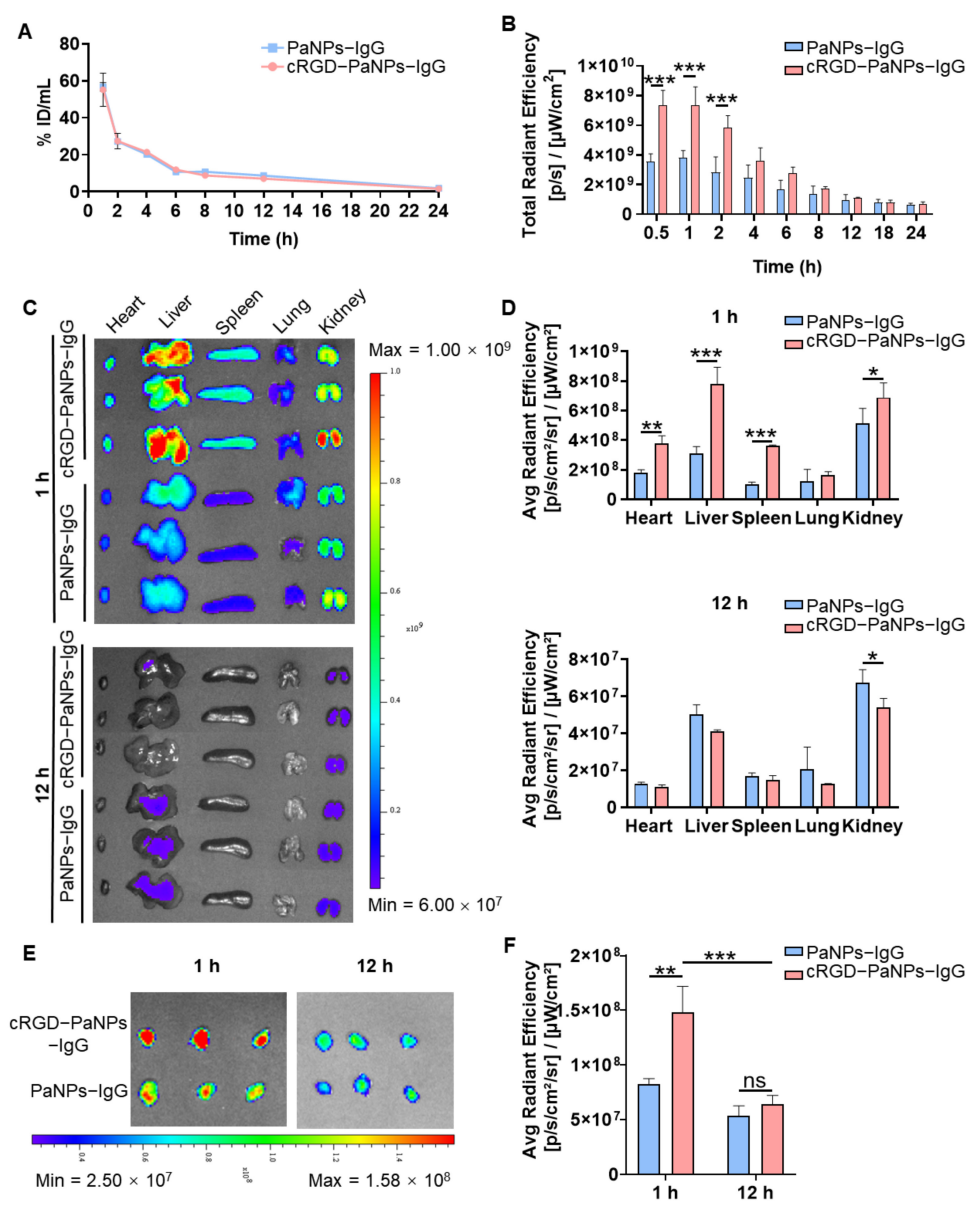
Biodistribution of Pa from PaNPs–IgG or cRGD–PaNPs–IgG in the 4T1–bearing mice. The mice were i.v. injected with PaNPs–IgG or cRGD–PaNPs–IgG (5 mg/kg of Pa, 5 mg/kg of IgG). (**A**) The blood concentration–time curves of Pa in healthy BALB/c mice. (**B**) The semi–quantitative analysis of fluorescence intensity of Pa in the tumor of mice. (**C**) Fluorescence images of main organs from 4T1–bearing mice at 1 h or 12 h after the injection. (**D**) Semi–quantitative analysis of the imaging results in (**C**). (**E**) Fluorescence images of the tumors at 1 h or 12 h after the i.v. injection. (**F**) Semi–quantitative analysis of the imaging results in (**D**). Data are expressed as mean ± SD (*n* = 3). * *p* < 0.05, ** *p* < 0.01 and *** *p* < 0.001. ns, no significant difference between the two compared groups.

The blood concentration–time curves of Pa in mice following the i.v. administration of PaNPs–IgG or cRGD–PaNPs–IgG were compared (Figure 4A and Supplementary Figure S3A). The half time (*t*_1/2_) of PaNPs–IgG and cRGD–PaNPs–IgG were 6.06 ± 0.96 and 6.30 ± 0.58 h, respectively, without a statistical significance (Table 1). cRGD–PaNPs–IgG exhibited similar pharmacokinetic parameters as PaNPs–IgG, suggesting that the modification with cRGD did not alter the pharmacokinetic properties of the liposomes.

The in vivo distribution of the liposomes and tumor targeting ability of PaNPs–IgG or cRGD–PaNPs–IgG were evaluated by IVIS. The results of the live imaging illustrated that the maximum fluorescence intensity in the tumor sites of mice was observed at 1 h post–injection of both liposomes, followed by a slow decrease over time (Figure 4B and Supplementary Figure S3B). The semi–quantitative analysis demonstrated that the fluorescence intensity at the tumor sites of the mice treated with the cRGD–PaNPs–IgG was approximately 2.08–fold at 0.5 h, 1.93–fold at 1 h and 2.07–fold at 2 h of that with PaNPs–IgG, respectively (Figure 4B). This was consistent with the results of the fluorescence micrographs (Supplementary Figure S3C) as an indicative of the tumor targeted delivery of cRGD–PaNPs–IgG to the overexpressed α_v_β_3_ of the tumor cells [31,36,37].

The analysis of the ex vivo samples of mice following the administration of the liposomes showed that the liver and kidney were major organs for the liposomal distribution (Figure 4C,D). In addition, the amount of Pa accumulated in the tumor of the cRGD–NPs–IgG–treated group was about 1.79–fold at 1 h of the PaNPs–IgG–treated group after the injection (Figure 4E,F), which was consistent with the in vivo imaging results. Accordingly, we chose the time point of 1 h post–injection for the PDT of the s.c. 4T1 tumor of mice.

### 3.9. The Antitumor Effect of PDT plus αPD–L1 by cRGD–PaNPs–αPD–L1

**Figure 5:**
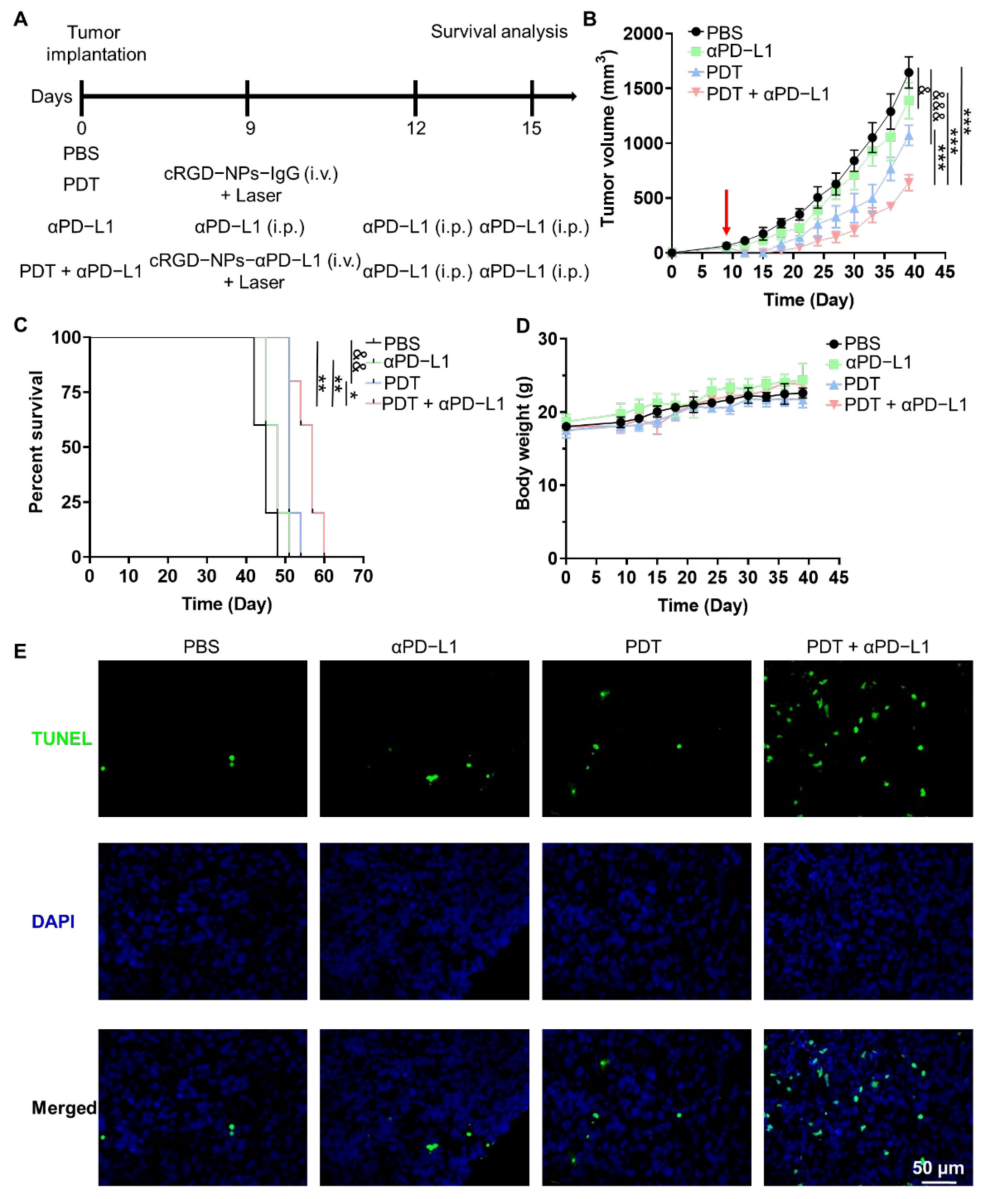
The antitumor effect of the combinatorial therapy of the 4T1–bearing mice. (**A**) Schematic illustration of the regimens for different treatments. (**B**) Average tumor growth curves over time. Arrow, treatment started. (**C**) Survival of the 4T1–bearing mice after the treatment. (**D**) Body weight of mice following the treatment. (**E**) Fluorescence micrographs of tumor stained with TUNEL following different treatment. Data are presented as mean ± SD (*n* = 5). * *p* < 0.05, ** *p* < 0.01 and *** *p* < 0.001 compared with the PDT + αPD–L1 group. ^&^ *p* < 0.05, ^&&^ *p* < 0.01 and ^&&&^ *p* < 0.001 compared with the PBS group.

The antitumor effect was carried out using mice bearing a 4T1 tumor following various treatments (Figure 5A). αPD–L1 showed a slightly inhibitory effect on the tumor compared with the PBS control. The inhibitory effect of PDT + αPD–L1 with cRGD–PaNPs–αPD–L1 on the 4T1 tumor was superior to the αPD–L1 group (Figure 5B and Supplementary Figure S4). The median survival time of the PDT + αPD–L1 group was 57 days, while that of the PBS group and αPD–L1 group were 45 days and 48 days, respectively. The results of the tumor growth curves demonstrated that the PDT + αPD–L1 treatment produced the most efficient anti–tumor effect among all the groups, resulting in the longest survival time of the mice (Figure 5C). The results of the TUNEL analysis further confirmed that the PDT + αPD–L1 treatment induced the highest amount of apoptotic or necrotic 4T1 tumor cells (Figure 5E). Meanwhile, all mice in the four groups did not exhibit any significant loss of their body weights (Figure 5D). These results collectively demonstrated a combined anti–tumor effect of PDT and αPD–L1 by cRGD–PaNPs–αPD–L1 with a low toxicity.

### 3.10. Enhanced Anti–Tumor Immunity by the Combinatorial Therapy with PDT and αPD–L1

**Figure 6:**
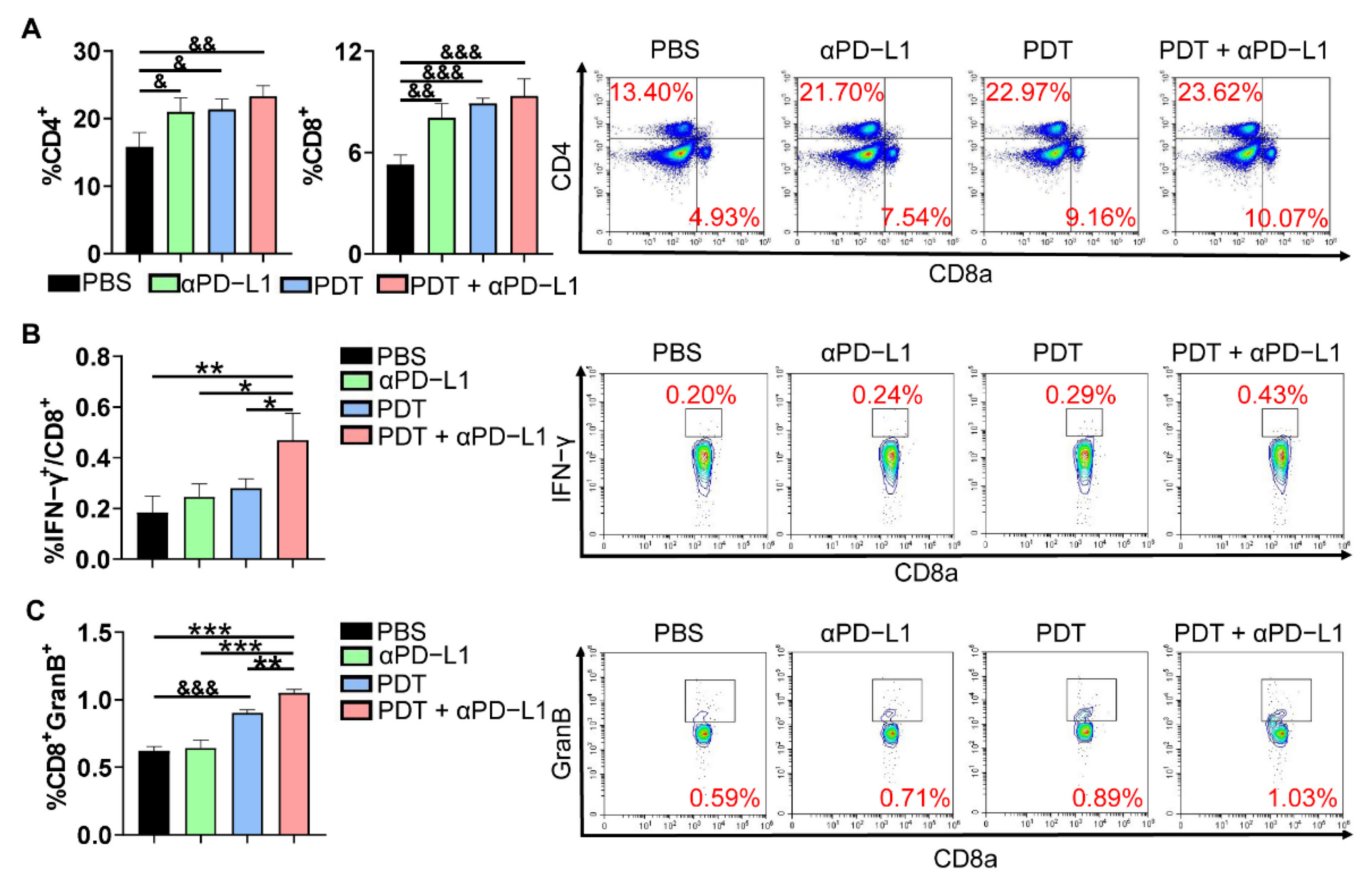
Flow cytometry analysis of various immune cells in the spleens in the 4T1–bearing mice following different treatment. (**A**) Percentage of CD4^+^ and CD8^+^ cells in spleens, (**B**) IFN–γ^+^ in CD8^+^ cells and (**C**) CD8^+^GranB^+^ in spleens, respectively. The therapeutic regimen was shown in Supplementary Figure S5. The representative flow cytometric histograms and their gating strategies are shown on the right. Data are presented as mean ± SD (*n* = 3). * *p* < 0.05, ** *p* < 0.01 and *** *p* < 0.001 compared with the PDT + αPD–L1 group. ^&^ *p* < 0.05, ^&&^ *p* < 0.01 and ^&&&^ *p* < 0.001 compared with the PBS group.

To analyze the immune response following the combinatorial photodynamic immunotherapy, spleens, DLNs and tumors were collected from the 4T1 tumor–bearing mice at 5 days after the last treatment according to the regimen shown in Supplementary Figure S5. In the spleens, all the three treatment groups increased the percentage of CD4^+^, CD8^+^, CTLs (CD8^+^IFN–γ^+^) and Granzyme B–positive CD8^+^ T cells (CD8^+^GranB^+^) compared with PBS (Figure 6). Additionally, the PDT + αPD–L1 group significantly increased the percentage of CD8^+^ cells from 5.28% to 9.36%, that of CTLs from 0.18% to 0.47% and that of the CD8^+^GranB^+^ T cells from 0.62% to 1.05% compared with the PBS group, suggesting that PDT combined with αPD–L1 induced a more significant antitumor effect.

**Figure 7:**
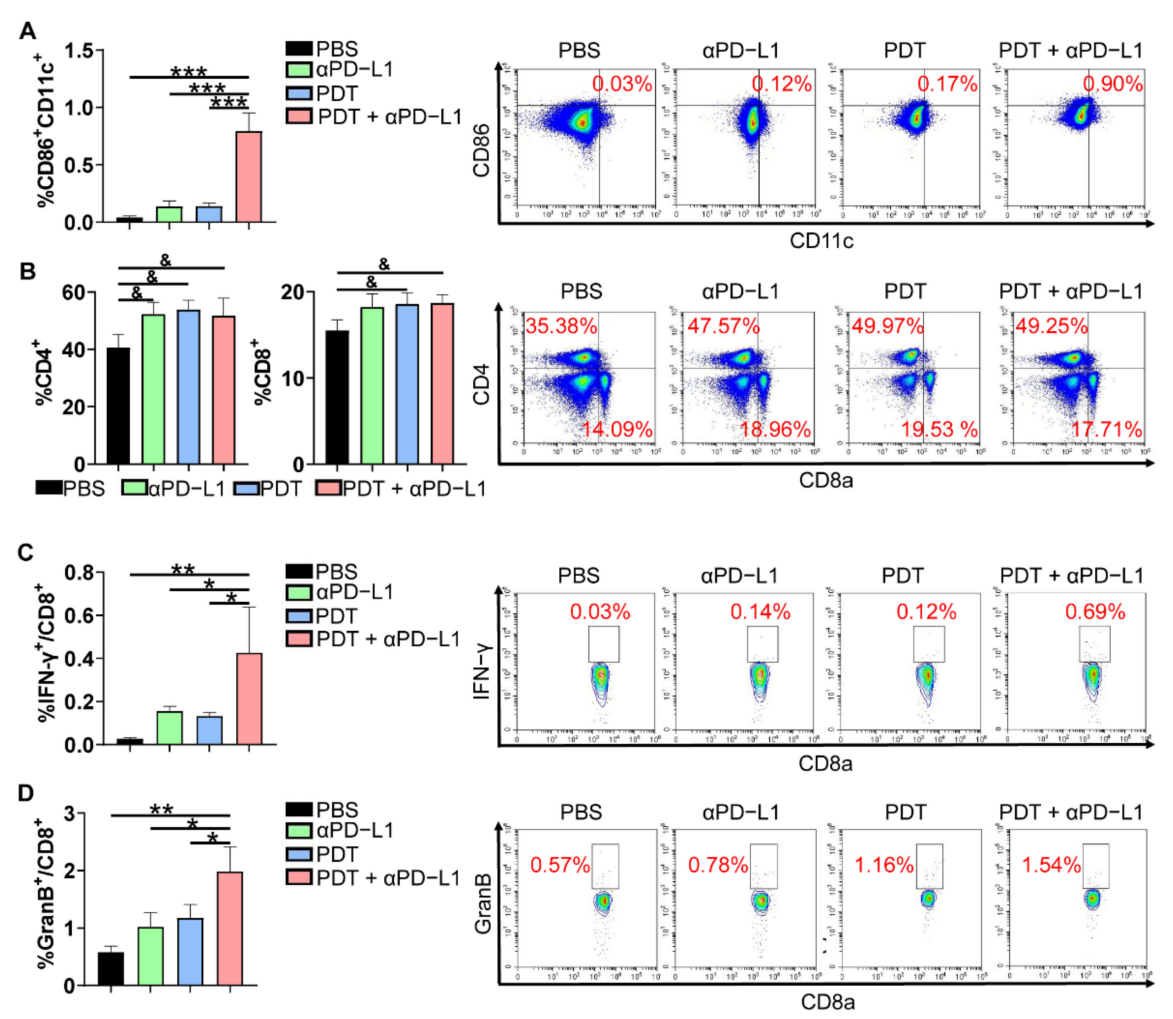
Flow cytometry analysis of various immune cells in the DLNs in the 4T1–bearing mice following different treatment. (**A**) Percentage of mDCs, (**B**) CD4^+^ and CD8^+^ cells, (**C**) IFN–γ^+^ in CD8^+^ cells, and (**D**) CD8^+^GranB^+^ cells in the DLNs, respectively. The therapeutic regimen was shown in Supplementary Figure S5. The representative flow cytometric histograms and their gating strategies are shown on the right. Data are presented as mean ± SD (*n* = 3). * *p* < 0.05, ** *p* < 0.01 and *** *p* < 0.001 compared with the PDT + αPD–L1 group. ^&^ *p* < 0.05 compared with the PBS group.

In DLNs, PDT + αPD–L1 induced the highest number of matured DCs (mDCs, CD11c^+^CD86^+^) among all the four treatment groups (Figure 7A). Furthermore, the amounts of the CD4^+^ and CD8^+^ cells were significantly increased following the treatment of PDT + αPD–L1 (Figure 7B). Specifically, the PDT + αPD–L1 group upregulated the percentages of CTLs from 0.03% to 0.43% and CD8^+^GranB^+^ T cells from 0.58% to 1.98% compared with the PBS group (Figure 7C,D).

**Figure 8:**
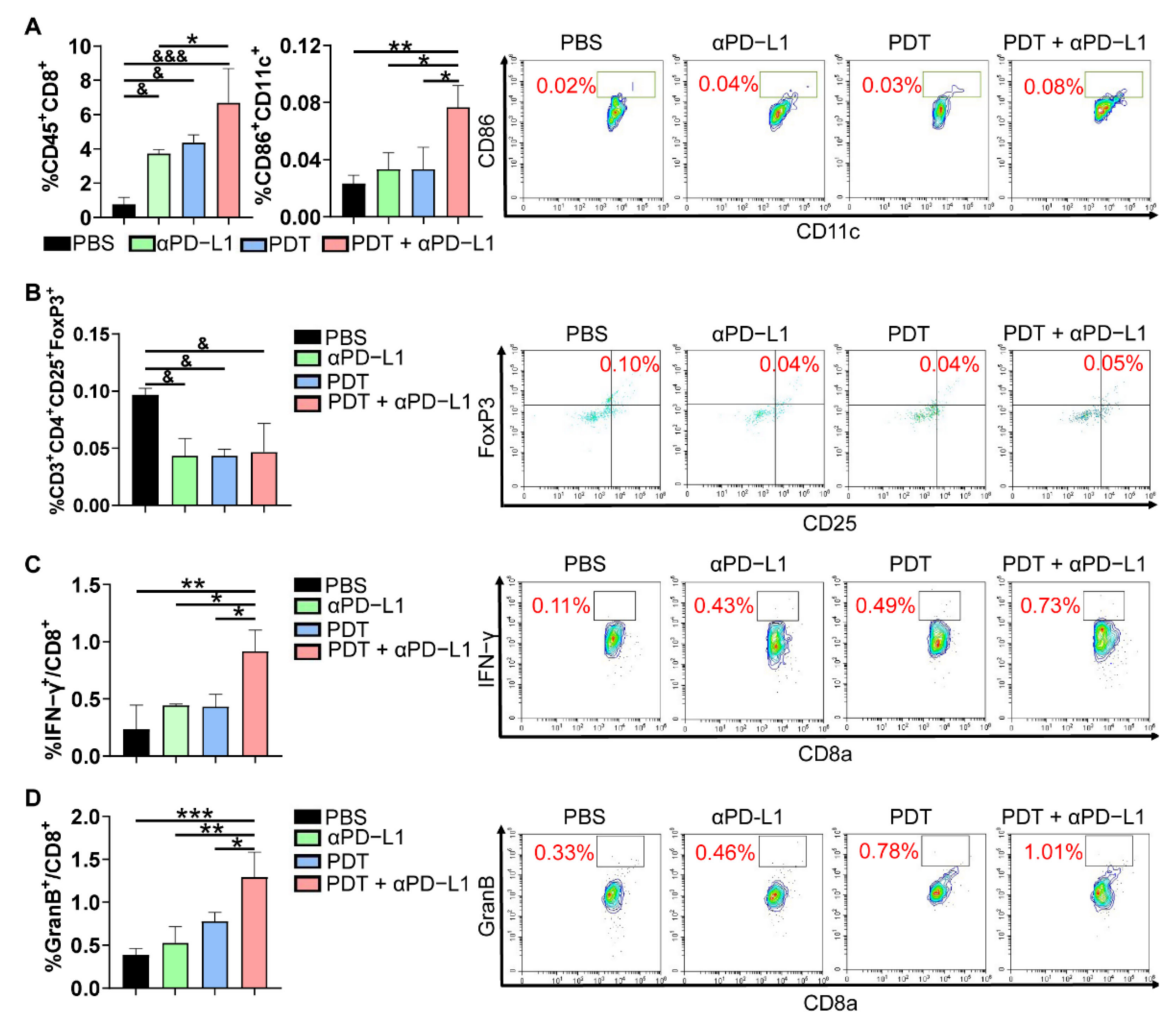
Flow cytometry analysis of various immune cells in the tumors following the treatment. Percentage of CD45^+^CD8^+^ cells and mDCs (**A**), Tregs (CD3^+^CD4^+^CD25^+^Foxp3^+^) (**B**), IFN–γ^+^ in CD8^+^ cells (**C**) and CD8^+^GranB^+^ cells (**D**) in the tumors. The therapeutic regimen was shown in Supplementary Figure S5. The representative flow cytometric histograms and their gating strategies are shown on the right. Data are presented as mean ± SD, *n* = 3. * *p* < 0.05, ** *p* < 0.01 and *** *p* < 0.001 compared with the PDT + αPD–L1 group. ^&^ *p* < 0.05 and ^&&&^ *p* < 0.001 compared with the PBS group.

The tumors were weighted before the flow cytometry experiments and the results were presented in the Supplementary Figure S6. In the tumors, the PDT + αPD–L1 treatment induced the highest amount of the tumor–infiltrating CD8^+^ T cells and mDCs among all the treatment groups (Figure 8A). The PDT + αPD–L1 group increased the percentages of the CD8^+^ cells from 0.76% to 6.70% and mDCs from 0.023% to 0.077% compared with the PBS group. Meanwhile, the PDT + αPD–L1 group significantly declined the regulatory T cells (Tregs, CD3^+^CD4^+^CD25^+^Foxp3^+^) from 0.097% to 0.047% compared with the PBS control (Figure 8B). This result suggested a promoted antitumor immunity by relieving the immune suppressive microenvironment. Furthermore, the significant inhibition of tumors treated with PDT + αPD–L1 was attributed to the increased infiltration of CTLs (CD8^+^IFN–γ^+^) and CD8^+^GranB^+^ T cells in the tumor (Figure 8C,D). The PDT + αPD–L1 group increased the percentages of CTLs from 0.23% to 0.92% and the CD8^+^GranB^+^ T cells from 0.39% to 1.29%. These results demonstrated that the treatment with PDT + αPD–L1 promoted the maturation and activation of DCs and activated the CTLs–mediated anti–tumor response for an efficient immunotherapy in addition to the PDT–mediated cell–killing effect.

### 3.11. cRGD–PaNPs–αPD–L1 with Good Biocompatibity

**Figure 9:**
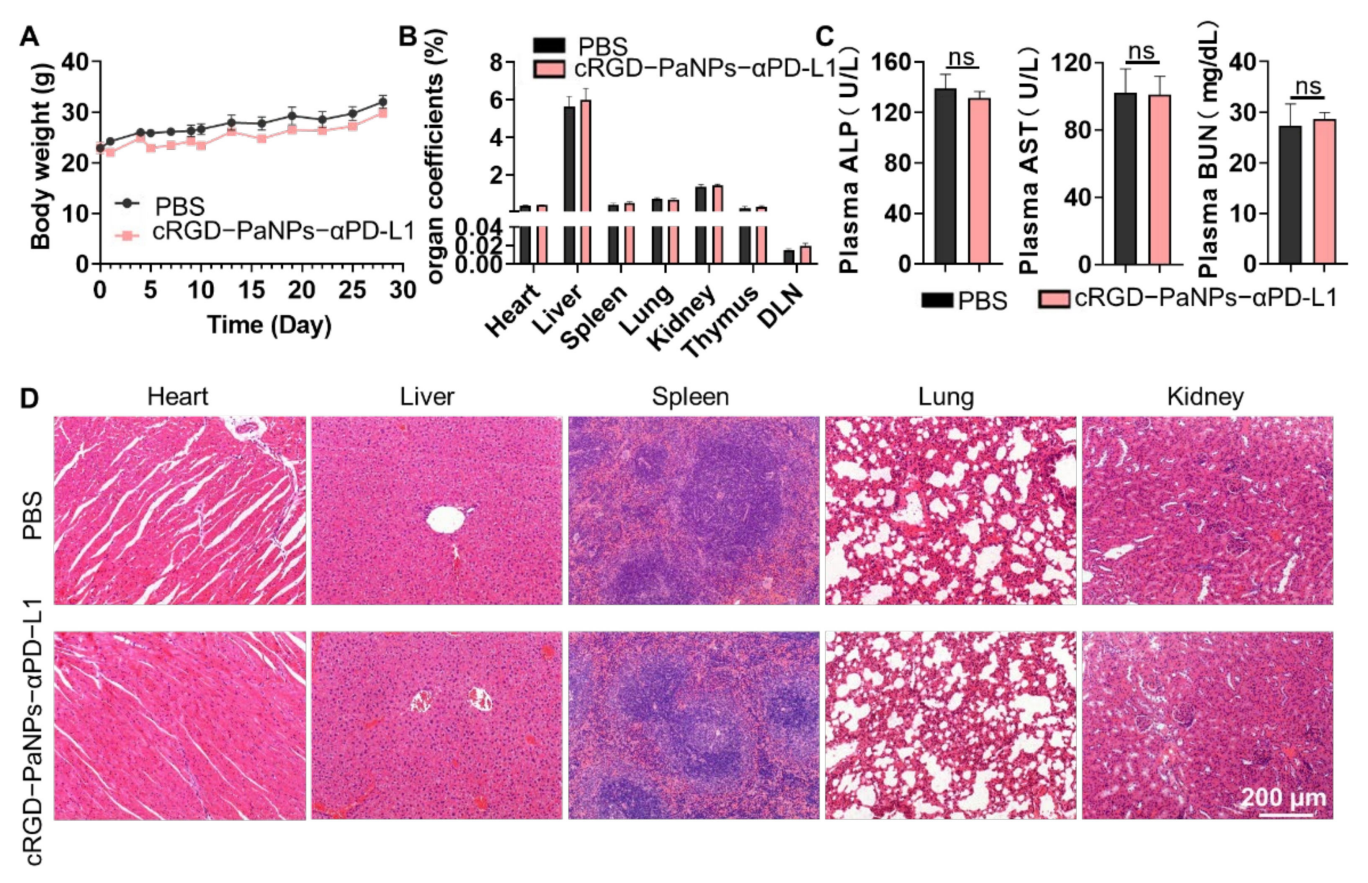
The toxicity evaluation of the cRGD–PaNPs–αPD–L1. (**A**) Body weight changes in the BALB/c mice after i.v. injection with cRGD–PaNPs (5 mg/kg of Pa and 100 µg of αPD–L1). (**B**) Organ coefficients. (**C**) ALP, AST and BUN levels in blood. (**D**) H&E staining of the major organs. Data are presented as mean ± SD (*n* = 3). ns, no significance between the two compared groups.

The systemic toxicity of mice after the combined treatment with the liposomal formulation was evaluated by measuring the change in the body weight, organ coefficient, biochemical indexes corresponding to the function of the liver and kidney and the morphology of the main organs by H&E staining. As shown in Figure 9A, no significant body weight loss was observed in the cRGD–PaNPs–αPD–L1 treatment group compared with the PBS control. Additionally, there were no evident pathological changes observed in the major organs. cRGD–PaNPs–αPD–L1 did not cause any significant changes in the organ coefficient compared with the PBS control (Figure 9B and Supplementary Figure S7). Alkaline phosphatase (ALP), aspartate aminotransferase (AST) and blood urea nitrogen (BUN) were within the normal ranges and not significantly changed in the cRGD–PaNPs–αPD–L1 treatment group compared with the PBS control (Figure 9C). In addition, the results of the H&E staining showed that there were no significant pathologic changes in the sections of major organs following the treatment (Figure 9D). The results of the whole blood chemistry showed that there was no significant change in the function of the liver or kidney following the treatment (Supplementary Table S4). Collectively, these results proved the good biocompatibility and safety of cRGD–PaNPs–αPD–L1 at the therapeutic dosage.

## 4. Discussion

Breast cancer often exhibited a poor response to the immunotherapy, including the immune checkpoint inhibitors. The Food and Drug Administration (FDA) only approved the atezolizumab in combination with the nab–paclitaxel for the patients with metastatic and inoperable triple–negative breast cancer (TNBC) with PD–L1 positively diagnosed [38]. Only about 12% of patients in the PD–L1–positive expression group and no patients (0%) in the PD–L1–negative expression group showed a clinical response to the anti–PD–L1 antibody [39]. Consequently, there is an urgent need to explore a more different adjuvant treatment to enhance the efficacy of PD–L1 for the TNBC patients with a low PD–L1 expression. By using the PD–L1–low expression breast tumor model, we showed that PDT by cRGD–PaNPs–IgG can elevate the PD–L1 level in tumor cells and promote the activation of mDCs and the infiltration of CTLs in the tumors. These results suggest a promising adjuvant strategy for the treatment of TNBC by enhancing the expression of PD–L1 in the tumors and reversing the immune suppressive tumor microenvironment to overcome the αPD–L1–irresponsiveness of TNBC.

## 5. Conclusions

In this work, we designed the tumor–targeted cRGD–PaNPs–αPD–L1 loading Pa and αPD–L1 for the combined PDT and PD–L1 blockade therapy of a 4T1 tumor with a low PD–L1 expression. cRGD–PaNPs–IgG were prepared by the reverse–phase evaporation method. The Pa–mediated PDT increased the PD–L1 expression of 4T1 tumor cells in vivo by 1.45–fold compared by the PBS control. The fluorescence intensity at the tumor site of the mice treated with the cRGD–PaNPs–IgG was 2.08–fold at 0.5 h, 1.93–fold at 1 h and 2.07–fold at 2 h of that with the PaNPs, respectively. This result demonstrated that cRGD–PaNPs–IgG improved the tumor accumulation of Pa. The combinatorial treatment with PDT and immunotherapy by cRGD–PaNPs–αPD–L1 (PDT + αPD–L1) delayed the tumor growth of the 4T1–bearing mice and prolonged the survival time (57 days) compared with the PBS control (45 days) or αPD–L1 group (48 days). The PDT + αPD–L1 group significantly ameliorated the immune environment of the 4T1 tumor of mice by promoting the activation of mDCs from 0.023% to 0.077% and enhancing the infiltration of CTLs from 0.23% to 0.92% and the CD8^+^GranB^+^ T cells from 0.39% to 1.29% compared with the PBS group. In addition, there was no significant difference between the cRGD–PaNPs–αPD–L1 and PBS group in the body weight, organ coefficient, or biochemical indexes corresponding to the function of the liver and kidney, indicating that the cRGD–PaNPs–αPD–L1 exerted a good biocompatibility with a negligible toxicity to normal organs. Taken together, our study presented a strategy for elevating the PD–L1 level in the tumor cells and suggested PDT to be a promising adjuvant treatment to the immune checkpoint blockade therapy of the tumor with a low PD–L1 expression.

## Figures and Tables

**Table 1:** Pharmacokinetic parameters of Pa after i.v. injection cRGD–PaNPs–IgG or PaNPs–IgG (5 mg/kg of Pa, 5 mg/kg of IgG) into female BALB/c mice ^a^.

Parameters ^b^	PaNPs–IgG	cRGD–PaNPs–IgG
*t*_1/2_ (h)	6.06 ± 0.96	6.30 ± 0.58
*k*	0.12 ± 0.017	0.11 ± 0.011
AUC_0→n_ (% ID/mL h)	257.90 ± 5.59	241.24 ± 11.47
AUMC (% ID/mL h^2^)	1772.10 ± 36.71	1552.34 ± 143.27
*Cl* (mL/h)	0.39 ± 0.0083	0.42 ± 0.020
MRT (h)	6.87 ± 0.099	6.42 ± 0.33
V*ss* (mL)	2.67 ± 0.082	2.66 ± 0.098

^a^ Data are presented as mean ± SD (*n* = 3). ^b^ *t*_1/2_, elimination half–life; *k*, elimination rate constant; AUC_0→n_, area under the concentration–time curve to the least measurable concentration; AUMC, area under moment curve; *Cl*, clearance; MRT, mean residence time; V*ss*, steady–state volume of distribution.

## Data Availability

The data presented in this study are available on request from the corresponding author.

## Supplementary Materials

The following supporting information can be downloaded at: https://www.mdpi.com/article/10.3390/pharmaceutics14112513/s1. Figure S1: The representative histograms of PD–L1 expression; Figure S2: Cellular uptake of different PaNPs; Figure S3: Pharmacokinetics and tumor distribution of Pa in BALB/c mice bearing 4T1 breast tumors; Figure S4: Individual tumor growth curves over time in each group of Figure 5B; Figure S5: The regimens of different treatment for the evaluation of anti–tumor immunity in mice bearing 4T1 breast tumors; Figure S6: Tumor weights and the photographs of the tumors of BALB/c mice bearing 4T1 breast cancer following the various treatment (*n* = 3); Figure S7: Photographs of the major organs and organ coefficients of mice at 28 days after i.v. injection of cRGD–PaNPs–αPD–L1 (5 mg/kg of Pa and 100 µg of αPD–L1) at days 0, 3 and 6; Table S1: Size distribution, PDI and zeta potential of PaNPs–IgG or cRGD–PaNPs–IgG; Table S2: The drug loading efficiency and encapsulation efficiency of Pa and IgG in PaNPs–IgG; Table S3: The drug loading efficiency and encapsulation efficiency of Pa and IgG in cRGD–PaNPs–IgG; Table S4: Blood chemistry and hematologic analysis of BALB/c mice; Table S5: Antibodies used for flow cytometry experiments in this study.

## References

[1] Yang Y. (2015). Cancer Immunotherapy: Harnessing the Immune System to Battle Cancer. J. Clin. Invest..

[2] Teng M.W., Ngiow S.F., Ribas A., Smyth M.J. (2015). Classifying Cancers Based on T-Cell Infiltration and Pd-L1. Cancer Res..

[3] He X., Xu C. (2020). Immune Checkpoint Signaling and Cancer Immunotherapy. Cell Res..

[4] Liu Y.T., Sun Z.J. (2021). Turning Cold Tumors into Hot Tumors by Improving T-Cell Infiltration. Theranostics.

[5] Tu J., Xu H., Ma L., Li C., Qin W., Chen X., Yi M., Sun L., Liu B., Yuan X. (2022). Nintedanib Enhances the Efficacy of Pd-L1 Blockade by Upregulating Mhc-I and Pd-L1 Expression in Tumor Cells. Theranostics.

[6] Xu J., Zheng Q., Cheng X., Hu S., Zhang C., Zhou X., Sun P., Wang W., Su Z., Zou T. (2021). Chemo-Photodynamic Therapy with Light-Triggered Disassembly of Theranostic Nanoplatform in Combination with Checkpoint Blockade for Immunotherapy of Hepatocellular Carcinoma. J. Nanobiotechnol..

[7] Grabovska Y., Mackay A., O’Hare P., Crosier S., Finetti M., Schwalbe E.C., Pickles J.C., Fairchild A.R., Avery A., Cockle J. (2020). Pediatric Pan-Central Nervous System Tumor Analysis of Immune-Cell Infiltration Identifies Correlates of Antitumor Immunity. Nat. Commun..

[8] Hudson K., Cross N., Jordan-Mahy N., Leyland R. (2020). The Extrinsic and Intrinsic Roles of Pd-L1 and Its Receptor Pd-1: Implications for Immunotherapy Treatment. Front. Immunol..

[9] Majidpoor J., Mortezaee K. (2021). The Efficacy of Pd-1/Pd-L1 Blockade in Cold Cancers and Future Perspectives. Clin. Immunol..

[10] Zhang Y., Zhang Z. (2020). The History and Advances in Cancer Immunotherapy: Understanding the Characteristics of Tumor-Infiltrating Immune Cells and Their Therapeutic Implications. Cell Mol. Immunol..

[11] Binnewies M., Roberts E.W., Kersten K., Chan V., Fearon D.F., Merad M., Coussens L.M., Gabrilovich D.I., Ostrand-Rosenberg S., Hedrick C.C. (2018). Understanding the Tumor Immune Microenvironment (Time) for Effective Therapy. Nat. Med..

[12] Yi M., Zheng X., Niu M., Zhu S., Ge H., Wu K. (2022). Combination Strategies with Pd-1/Pd-L1 Blockade: Current Advances and Future Directions. Mol. Cancer..

[13] Kordbacheh T., Honeychurch J., Blackhall F., Faivre-Finn C., Illidge T. (2018). Radiotherapy and Anti-Pd-1/Pd-L1 Combinations in Lung Cancer: Building Better Translational Research Platforms. Ann. Oncol..

[14] Xiang Q., Yang C., Luo Y., Liu F., Zheng J., Liu W., Ran H., Sun Y., Ren J., Wang Z. (2022). Near-Infrared Ii Nanoadjuvant-Mediated Chemodynamic, Photodynamic, and Photothermal Therapy Combines Immunogenic Cell Death with Pd-L1 Blockade to Enhance Antitumor Immunity. Small.

[15] Wang M., Rao J., Wang M., Li X., Liu K., Naylor M.F., Nordquist R.E., Chen W.R., Zhou F. (2021). Cancer Photo-Immunotherapy: From Bench to Bedside. Theranostics.

[16] Donohoe C., Senge M.O., Arnaut L.G., Gomes-da-Silva L.C. (2019). Cell Death in Photodynamic Therapy: From Oxidative Stress to Anti-Tumor Immunity. Biochim. Biophys. Acta Rev. Cancer.

[17] Feng B., Hou B., Xu Z., Saeed M., Yu H., Li Y. (2019). Self-Amplified Drug Delivery with Light-Inducible Nanocargoes to Enhance Cancer Immunotherapy. Adv. Mater..

[18] Li W., Yang J., Luo L., Jiang M., Qin B., Yin H., Zhu C., Yuan X., Zhang J., Luo Z. (2019). Targeting Photodynamic and Photothermal Therapy to the Endoplasmic Reticulum Enhances Immunogenic Cancer Cell Death. Nat. Commun..

[19] Yuan Z., Fan G., Wu H., Liu C., Zhan Y., Qiu Y., Shou C., Gao F., Zhang J., Yin P. (2021). Photodynamic Therapy Synergizes with Pd-L1 Checkpoint Blockade for Immunotherapy of Crc by Multifunctional Nanoparticles. Mol. Ther..

[20] Ruiz-González R., Milán P., Bresolí-Obach R., Stockert J.C., Villanueva A., Cañete M., Nonell S. (2017). Photodynamic Synergistic Effect of Pheophorbide a and Doxorubicin in Combined Treatment against Tumoral Cells. Cancers.

[21] Tang P.M., Bui-Xuan N.H., Wong C.K., Fong W.P., Fung K.P. (2010). Pheophorbide a-Mediated Photodynamic Therapy Triggers Hla Class I-Restricted Antigen Presentation in Human Hepatocellular Carcinoma. Transl. Oncol..

[22] Aksel M., Bozkurt-Girit O., Bilgin M.D. (2020). Pheophorbide a-Mediated Sonodynamic, Photodynamic and Sonophotodynamic Therapies against Prostate Cancer. Photodiagnosis Photodyn. Ther..

[23] Xodo L.E., Rapozzi V., Zacchigna M., Drioli S., Zorzet S. (2012). The Chlorophyll Catabolite Pheophorbide a as a Photosensitizer for the Photodynamic Therapy. Curr. Med. Chem..

[24] Zhang Z., Wang K., Liu M., Hu P., Xu Y., Yin D., Yang Y., Dong X., Qu C., Zhang L. (2022). Phototherapeutic Effect of Transformable Peptides Containing Pheophorbide a on Colorectal Cancer. Drug Deliv..

[25] Chen J., Zhu Y., Wu C., Shi J. (2020). Nanoplatform-Based Cascade Engineering for Cancer Therapy. Chem. Soc. Rev..

[26] Luo L., Qi Y., Zhong H., Jiang S., Zhang H., Cai H., Wu Y., Gu Z., Gong Q., Luo K. (2022). Gsh-Sensitive Polymeric Prodrug: Synthesis and Loading with Photosensitizers as Nanoscale Chemo-Photodynamic Anti-Cancer Nanomedicine. Acta Pharm. Sin. B.

[27] Kim D.H., Im B.N., Hwang H.S., Na K. (2018). Gemcitabine-Loaded Dspe-Peg-Pheoa Liposome as a Photomediated Immune Modulator for Cholangiocarcinoma Treatment. Biomaterials.

[28] Lamch Ł., Pucek A., Kulbacka J., Chudy M., Jastrzębska E., Tokarska K., Bułka M., Brzózka Z., Wilk K.A. (2018). Recent Progress in the Engineering of Multifunctional Colloidal Nanoparticles for Enhanced Photodynamic Therapy and Bioimaging. Adv. Colloid. Interface Sci..

[29] Desgrosellier J.S., Cheresh D.A. (2010). Integrins in Cancer: Biological Implications and Therapeutic Opportunities. Nat. Rev. Cancer.

[30] Fu D., Huang X., Lv Z., Zhang Y., Chen M., Zhang W., Su D. (2022). Ultrasound and Magnetic Resonance Imaging of Cyclic Arginine Glycine Aspartic Acid-Gadopentetic Acid-Polylactic Acid in Human Breast Cancer by Targeting Avβ3 in Xenograft-Bearing Nude Mice. Bioengineered.

[31] Liu J., Deng H., Liu Q., Chu L., Zhang Y., Yang C., Zhao X., Huang P., Deng L., Dong A. (2015). Integrin-Targeted Ph-Responsive Micelles for Enhanced Efficiency of Anticancer Treatment in Vitro and in Vivo. Nanoscale.

[32] Yang S., Wang D., Zhang X., Sun Y., Zheng B. (2021). Crgd Peptide-Conjugated Polyethylenimine-Based Lipid Nanoparticle for Intracellular Delivery of Sirna in Hepatocarcinoma Therapy. Drug Deliv..

[33] Wu A., Chen Y., Wang H., Chang Y., Zhang M., Zhao P., Tang Y., Xu Q., Zhu Z., Cao Y. (2021). Genetically-Engineered “All-in-One” Vaccine Platform for Cancer Immunotherapy. Acta Pharm. Sin. B.

[34] Xu J., Yu S., Wang X., Qian Y., Wu W., Zhang S., Zheng B., Wei G., Gao S., Cao Z. (2019). High Affinity of Chlorin E6 to Immunoglobulin G for Intraoperative Fluorescence Image-Guided Cancer Photodynamic and Checkpoint Blockade Therapy. ACS Nano.

[35] Hu Z., Zheng B., Xu J., Gao S., Lu W. (2020). An Albumin-Bound Drug Conjugate of Paclitaxel and Indoleamine-2,3-Dioxygenase Inhibitor for Enhanced Cancer Chemo-Immunotherapy. Nanotechnology.

[36] Lee E., Park J., Youn Y.S., Oh K.T., Kim D., Lee E.S. (2020). Alendronate/Crgd-Decorated Ultrafine Hyaluronate Dot Targeting Bone Metastasis. Biomedicines.

[37] Nieberler M., Reuning U., Reichart F., Notni J., Wester H.J., Schwaiger M., Weinmüller M., Räder A., Steiger K., Kessler H. (2017). Exploring the Role of Rgd-Recognizing Integrins in Cancer. Cancers.

[38] Kwapisz D. (2021). Pembrolizumab and Atezolizumab in Triple-Negative Breast Cancer. Cancer Immunol. Immunother..

[39] Wu S., Shi X., Wang J., Wang X., Liu Y., Luo Y., Mao F., Zeng X. (2021). Triple-Negative Breast Cancer: Intact Mismatch Repair and Partial Co-Expression of Pd-L1 and Lag-3. Front. Immunol..

