# Supplementary material for: Pheophorbide A–Mediated Photodynamic Therapy Potentiates Checkpoint Blockade Therapy of Tumor with Low PD–L1 Expression
Source: Pharmaceutics. 2022 Nov 18;14(11):2513. doi: 10.3390/pharmaceutics14112513 (PMC9697200; doi:10.3390/pharmaceutics14112513)
Supplement: Supplementary file 1 [file pharmaceutics-14-02513-s001.zip › pharmaceutics-2020559-supplementary.pdf]

Supplementary materials

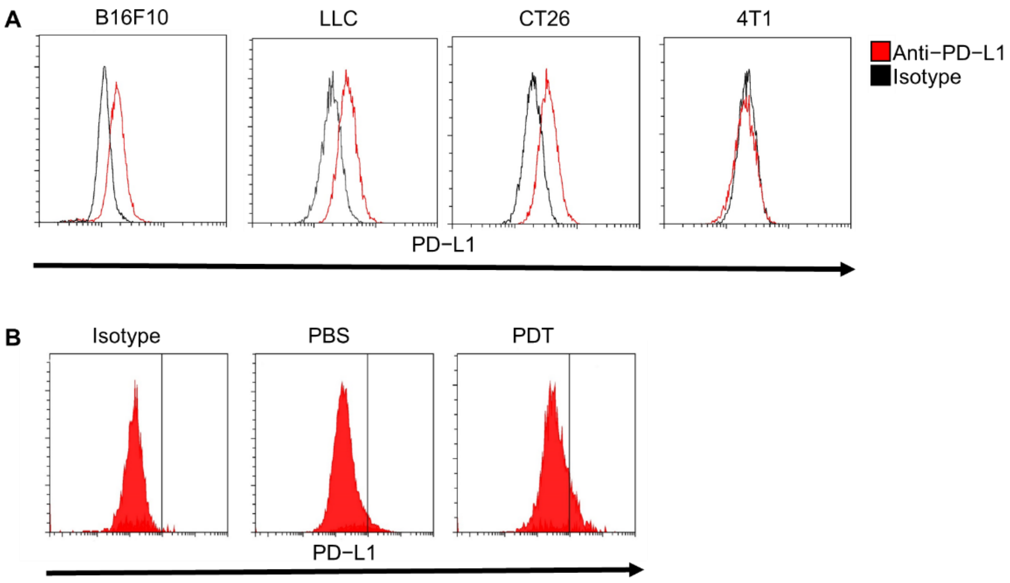

Figure S1. The representative histograms of PD−L1 expression. (A) The representative histograms of the result in Figure 2A. (B) The representative histograms of the result in Figure 2B.

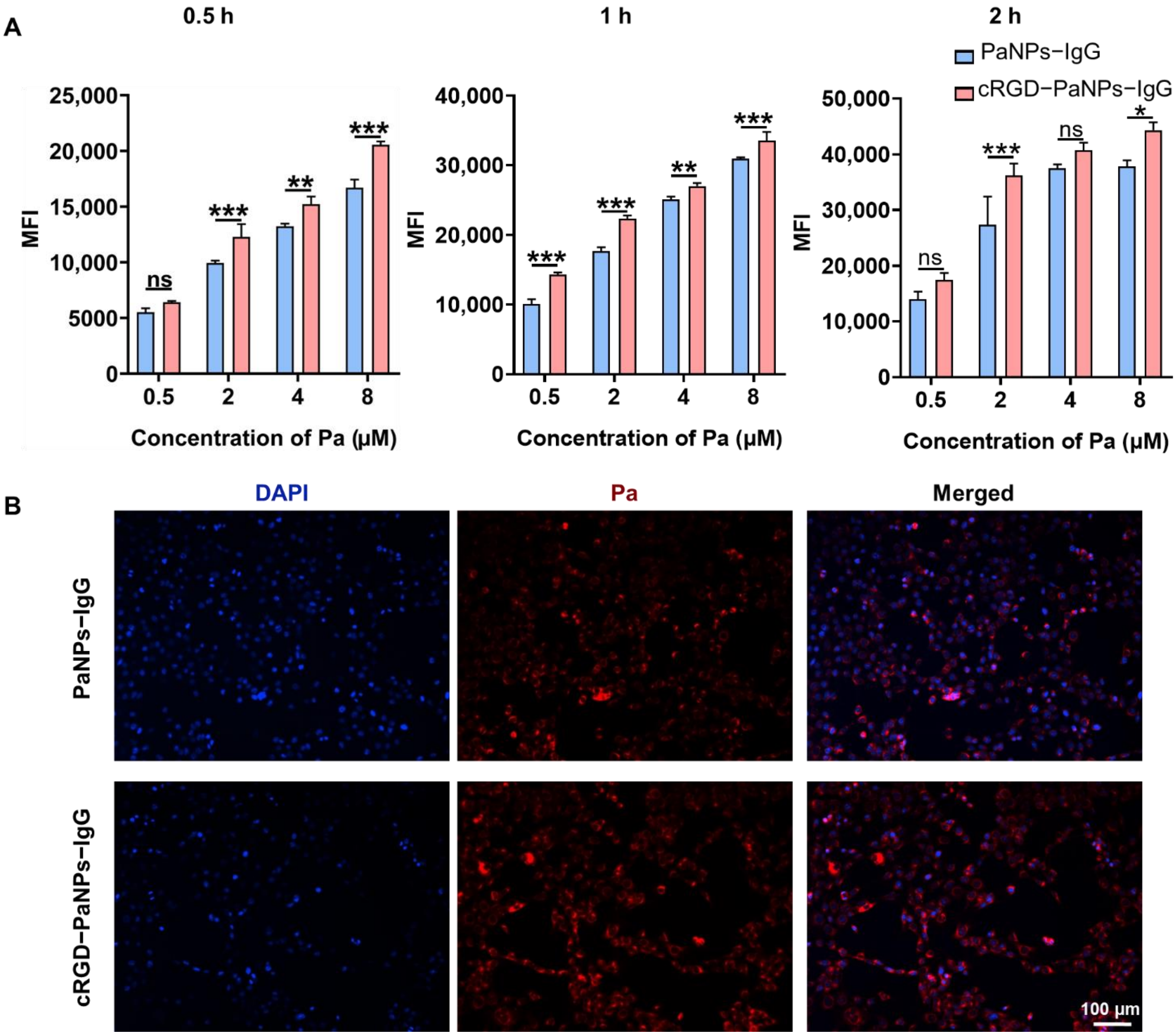

Figure S2. Cellular uptake of different PaNPs. (**A**) Quantitative analysis of cellular uptake measured by flow cytometry after incubation with different concentration of PaNPs−IgG or cRGD−PaNPs−IgG (n = 3). (**B**) Fluorescence micrographs of 4T1 cells after the incubation with PaNPs−IgG or cRGD−PaNPs−IgG (4 μM of Pa) for 2 h. Data are presented as mean ± SD. *P<0.05, **P<0.01, ***P<0.001. ns, no significant difference between the two compared groups.

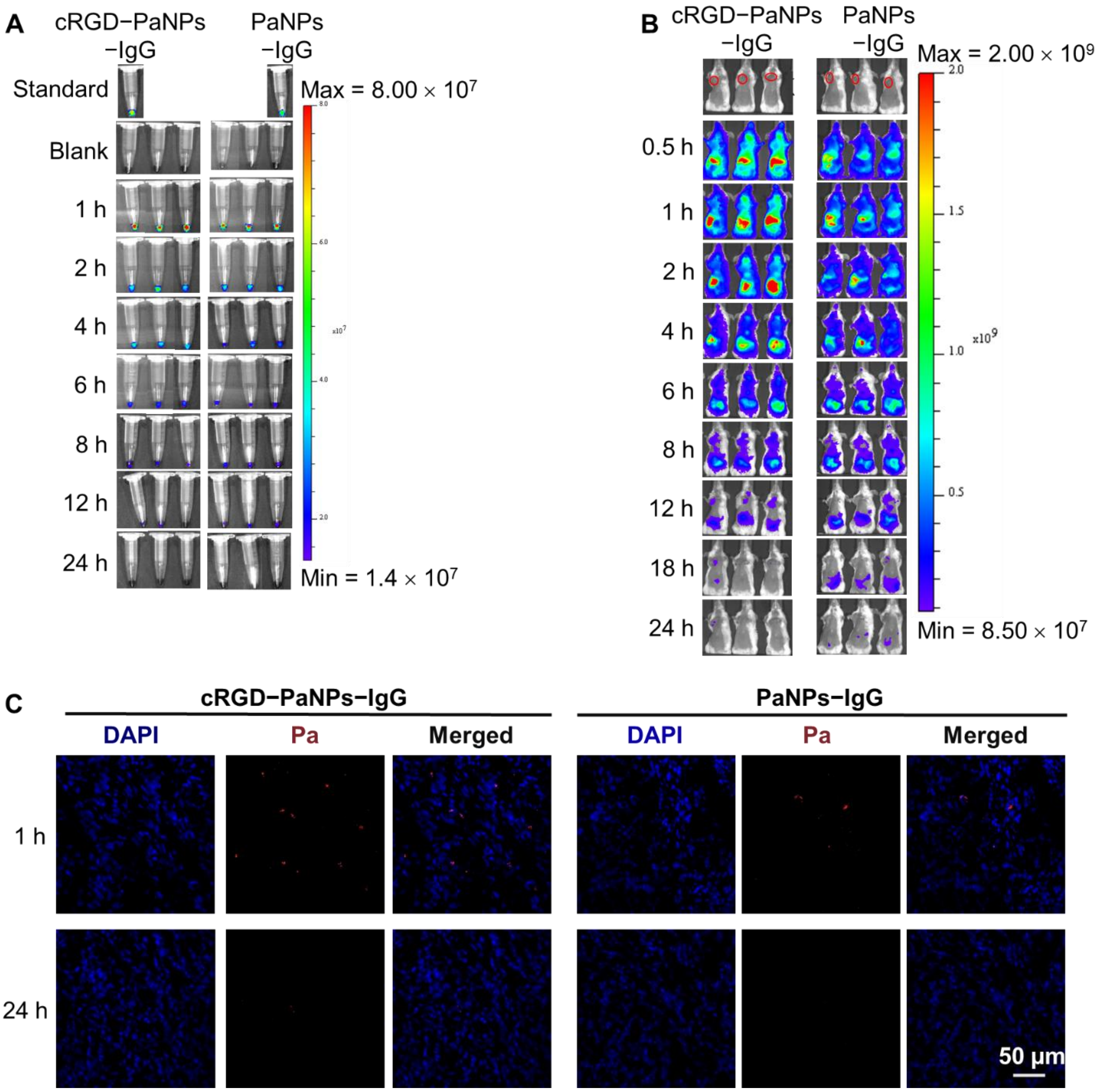

Figure S3. Pharmacokinetics and tumor distribution of Pa in BALB/c mice bearing 4T1 breast tumors. (**A**) Fluorescence images of the blood samples collected at different time points after i.v. injection of PaNPs−IgG or cRGD−PaNPs−IgG (5 mg/kg of Pa, 5 mg/kg of IgG). (**B**) Fluorescence images of the mice following the injection. (**C**) Confocal laser scanning micrographs of the tumor sections at 1 h or 24 h after the injection.

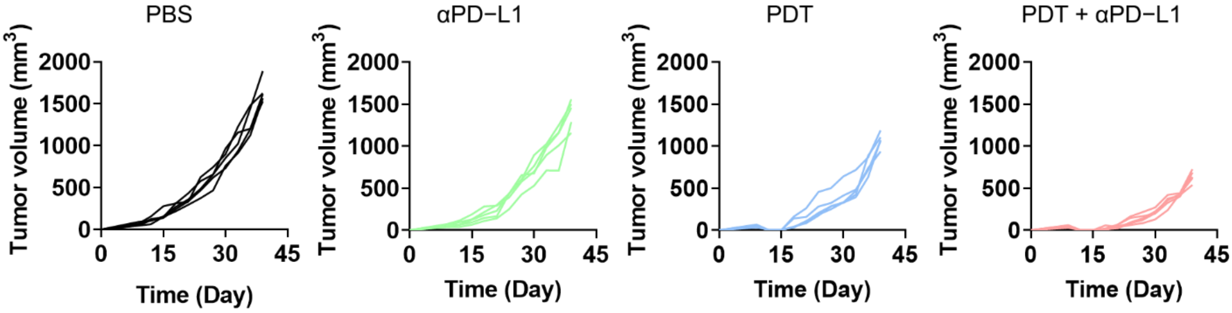

Figure S4. Individual tumor growth curves over time in each group of Figure 5B.

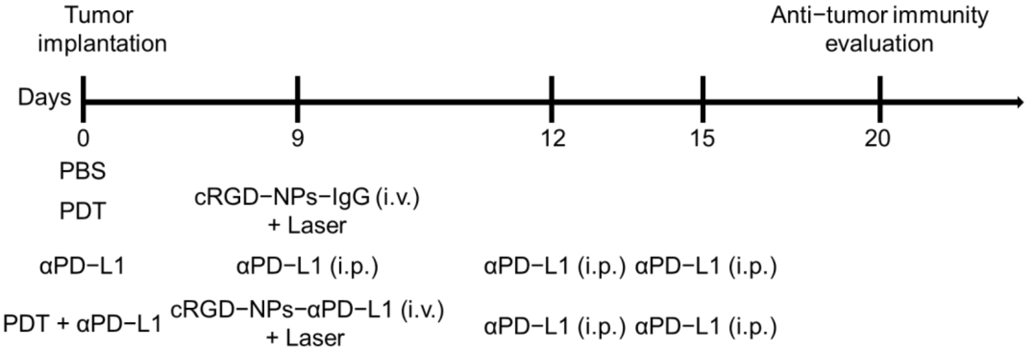

Figure S5. The regimens of different treatment for the evaluation of anti−tumor immunity in mice bearing 4T1 breast tumors. The tumors, spleens and draining lymph nodes (DLNs) were collected and evaluated from the four groups at 5 days after the last injection.

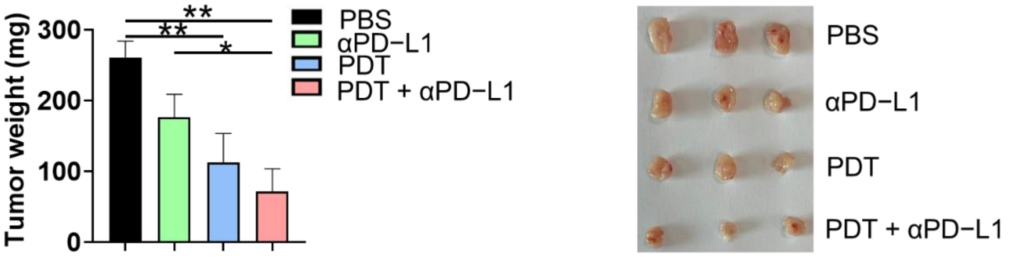

Figure S6. Tumor weights and the photographs of the tumors of BALB/c mice bearing 4T1 breast cancer following the various treatment (n = 3). The treatment regimens were shown in Supplementary Figure S5. The tumors were collected, weighted and photographed at 5 days after the last treatment. Data are presented as mean ± SD. *P<0.05 and **P<0.01.

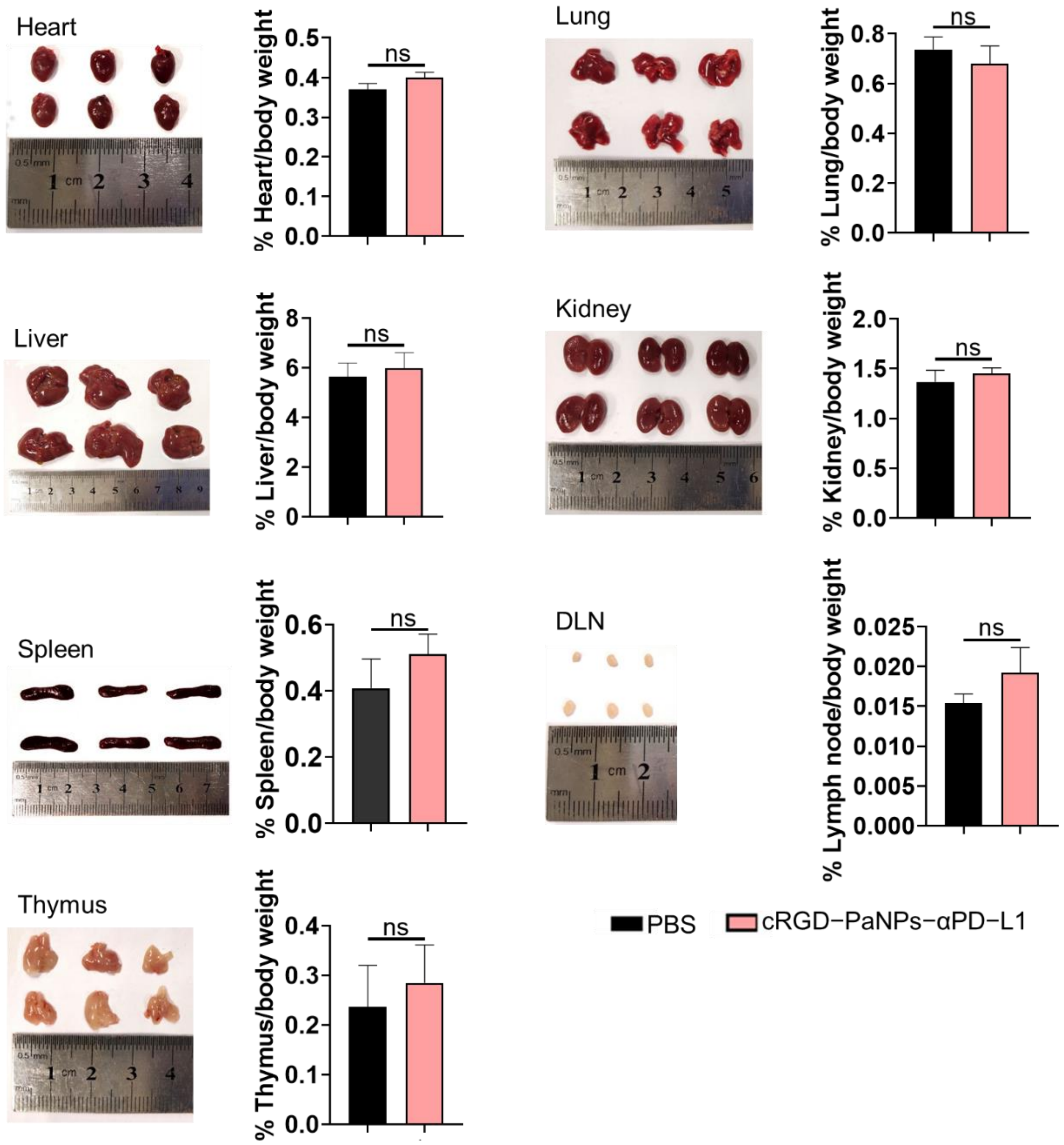

Figure S7. Photographs of the major organs and organ coefficients of mice at 28 days after i.v. injection of cRGD−PaNPs−αPD−L1 (5 mg/kg of Pa and 100 μg of αPD−L1) at days 0, 3 and 6. Mice treated with PBS solution were used as control (n = 3). Data are presented as mean ± SD. ns, no significant difference between the two compared groups.

Table S1. Size distribution, PDI and zeta potential of PaNPs−IgG or cRGD−PaNPs−IgG [a].

	**PaNPs−IgG**	**cRGD−PaNPs−IgG**
Particle mean size (nm)	173.70 ± 3.32	178.00 ± 3.89
PDI	0.19 ± 0.028	0.214 ± 0.06
Zeta potential (mV)	−7.88 ± 0.046	−10.20 ± 0.45

[a] Data are presented as mean ± SD (n = 3).

Table S2. The drug loading efficiency and encapsulation efficiency of Pa and IgG in PaNPs−IgG [a].

	**Pa**	**IgG**
Drug loading (%)	1.63 ± 0.034	1.96 ± 0.031
Encapsulation efficiency (%)	85.07 ± 1.76	89.99 ± 1.48

[a] Data are presented as mean ± SD (n = 3).

Table S3. The drug loading efficiency and encapsulation efficiency of Pa and IgG in cRGD−PaNPs−IgG [a].

	**Pa**	**IgG**
Drug loading (%)	1.40 ± 0.023	1.52 ± 0.0021
Encapsulation efficiency (%)	89.80 ± 1.45	88.03 ± 0.12

[a] Data are presented as mean ± SD (n = 3).

Table S4. Blood chemistry and hematologic analysis of BALB/c mice [a].

Items	Reference range	PBS	cRGD−PaNPs−αPD−L1
WBC (10^9/L)	5.69−14.84	6.23 ± 0.34	6.63 ± 0.69
Lymph (10^9/L)	3.6−11.56	4.03 ± 1.88	4.83 ± 1.30
MONO (10^9/L)	0.34−1.37	0.36 ± 0.09	0.43 ± 0.05
NEUT (10^9/L)	0.74−3.01	0.77 ± 0.05	2.30 ± 1.34
LYMPH (%)	55.06−83.82	76.87 ± 2.76	83.80 ± 3.14
MONO (%)	3.75−14.33	6.47 ± 1.54	9.30 ± 3.68
NEUT%	10.39−27.88	16.67 ± 1.27	12.17 ± 2.39
RBC (10^{12}/L)	8.16−11.69	9.83 ± 0.68	10.57 ± 0.42
HGB (g/dL)	12.4−18.9	15.93 ± 1.01	16.20 ± 0.51
HCT (%)	43.5−67.0	56.03 ± 2.94	57.50 ± 2.02
MCV (fL)	50.8−64.1	57.13 ± 2.94	54.43 ± 0.91
MCH (pg)	13.0−17.6	16.17 ± 0.24	15.30 ± 0.92
MCHC (g/dL)	23.9−33.1	28.37 ± 0.41	28.13 ± 0.12
RDW (%)	16.9−23.5	16.57 ± 0.46	17.50 ± 0.65
PLT (10^9/L)	476−1611	916.33 ± 73.83	1006.00 ± 74.26
MPV (fL)	4.6−5.8	5.80 ± 0.22	5.43 ± 0.17
ALP (U/L)	22.5−474.35	138.91 ± 9.11	131.12 ± 4.38
AST (U/L)	67−381	102.11 ± 11.58	100.77 ± 8.96
BUN (mg/dL)	7−31	2.28 ± 3.54	28.66 ± 1.04

[a] Blood chemistry and hematologic analysis of BALB/c mice at 28 days after i.v. injection of cRGD−PaNPs−αPD−L1 (5 mg/kg of Pa and 100 μg of αPD−L1) at days 0, 3 and 6. Mice treated with PBS solution were used as control (n = 3). Reference range of hematology data of healthy female BALB/c mice were obtained from Charles River Laboratories: (http://www.criver.com/). Data are presented as mean ± SD.

Table S5. Antibodies used for flow cytometry experiments in this study.

	Protein	Color	Clone	Item No.	Supplier
1	CD45	Pe−Cy5	30−F11	15−0451−82	eBioscience
2	CD8	FITC	53−6.7	11−0081−85	eBioscience
3	IFN−γ	APC	XMG1.2	17−7311−82	eBioscience
4	Rat IgG1 kappa	APC	eBRG1	17−4301−81	eBioscience
5	Granzyme B	PE	NGZB	12−8898−82	eBioscience
6	Rat IgG2a kappa	PE	eBR2a	12−4321−80	eBioscience
7	CD11c	PerCP−Cy5.5	N418	45−0114−82	eBioscience
8	CD86	FITC	GL1	11−0862−82	eBioscience
9	MHC−II	APC	I−A/I−E	17−5321−82	eBioscience
10	CD3	FITC	145−2C11	11−0031−82	eBioscience
11	CD25	PE	PC61.5	12−0251−82	eBioscience
12	CD4	APC	GK1.5	17−0041−82	eBioscience
13	Foxp3	PerCP−Cy5.5	FJK−16s	45−5773−82	eBioscience
14	PD−L1	PE	MIH5	12−5982−81	eBioscience
